# Tumor cells promote immunosuppression in ovarian cancer via a positive feedback loop with MDSCs through the SAA1–IL-1β axis

**DOI:** 10.1186/s13046-025-03536-y

**Published:** 2025-09-30

**Authors:** Haoran Hu, Meiqin Yang, Baoyou Huang, Jianyi Ding, Yashi Zhu, Xinxin Xu, Bo Yin, Huijuan Zhou, Tiefeng Huang, Mengjie Li, Yifan Kou, Zilale Rahim, Ang Li, Wei Wang, Lingfei Han

**Affiliations:** 1 Department of Gynecology, Shanghai Key Laboratory of Maternal Fetal Medicine, Shanghai Institute of Maternal-Fetal Medicine and Gynecologic Oncology, Shanghai First Maternity and Infant Hospital, School of Medicine, Tongji University, Shanghai, 200092 China; 2 Department of Gynecology, Shanghai Tenth People’s Hospital, Tongji University School of Medicine, Shanghai, China; 3 School of life science and technology, Tongji University, Shanghai, China; 4 Department of Gynecology, The First Affiliated Hospital of Wenzhou Medical University, Wenzhou, Zhejiang, 325027 China

**Keywords:** SAA1, MDSCs, IL-1β, Toll-like receptor, Immunosuppressive microenvironment

## Abstract

**Background:**

Immune tolerance in epithelial ovarian cancer (EOC) enables cancer cells to evade immune surveillance. Myeloid-derived suppressor cells (MDSCs), as crucial immunosuppressive regulators, shape the tumor microenvironment and contribute to resistance against immunotherapy. However, the regulatory mechanisms of MDSCs in ovarian cancer remain poorly understood.

**Methods:**

We examined the presence and distribution of MDSCs in peripheral blood and tumor tissues from EOC patients. Transcriptomic analysis was performed on ovarian cancer cells co-cultured with MDSCs. The role of Serum Amyloid A1 (SAA1) was investigated through in vitro functional assays, co-culture experiments, and in vivo mouse models.

**Results:**

MDSCs were enriched in both peripheral blood and tumor tissues of EOC patients. SAA1 was significantly upregulated in ovarian cancer cells after interaction with MDSCs and confirmed in tumor samples and cell lines. Functionally, SAA1 promoted cancer cell proliferation, migration, and invasion. It also recruited MDSCs via TLR2/4, induced the differentiation of granulocyte-monocyte progenitors (GMPs), and stimulated IL-1β secretion, which in turn enhanced SAA1 expression, forming a positive feedback loop. In vivo, SAA1 promoted tumor progression and ascites formation. Clinically, high levels of SAA1, IL-1β, and CD33⁺ MDSCs correlated with poor survival.

**Conclusion:**

This study uncovers a novel SAA1–IL-1β feedback loop that promotes immunosuppression and progression in ovarian cancer. These findings provide insight into tumor–immune interactions and suggest a potential biomarker and therapeutic target for EOC.

**Supplementary Information:**

The online version contains supplementary material available at 10.1186/s13046-025-03536-y.

## Introduction

Epithelial ovarian cancer (EOC) is recognized as the most lethal gynecological malignancy, with an annual global incidence of 324,398 new cases and 206,839 deaths [1]. The absence of effective screening methods leads to the diagnosis of most patients at an advanced stage. While initial surgery and chemotherapy yield favorable responses in EOC patients, the development of drug resistance and relapse is common. Notably, over 70% of patients ultimately experience relapse, resulting in a poor five-year survival rate of less than 30% [2, 3]. Given the persistent challenge of enhancing the long-term survival of EOC patients, there is an imperative need to explore the fundamental cellular and molecular mechanisms driving tumor progression and metastasis in these patients. This exploration, from a novel perspective, aims to provide insights and potential breakthroughs for the clinical treatment of EOC.

Immunotherapy, as an emerging therapeutic approach, has demonstrated notable efficacy across various malignant tumors. However, the clinical application of immunotherapy, particularly with PD-1/PD-L1 immune checkpoint inhibitors, remains limited in ovarian cancer [4]. This limitation is attributed to diverse immunosuppressive mechanisms within the tumor microenvironment (TME). In advanced ovarian cancer patients, the substantial tumor burden amplifies immunosuppressive signals in the TME, curtailing immune responses and impeding sensitivity to immunotherapy [5]. Myeloid-derived suppressor cells (MDSCs), recognized as the “queen bee” of the tumor-promoting immunosuppressive microenvironment, exhibit potent immunosuppressive functions [6–8]. In healthy individuals, immature myeloid cells (IMCs) differentiate into granulocytes, macrophages, and dendritic cells, contributing to normal immune functions within corresponding organs and tissues [9]. Under pathological conditions, such as cancer, infectious diseases, autoimmune disorders, or sepsis, the aberrant differentiation of normal myeloid lineages and sustained stimulation of myelopoiesis contribute to the expansion of MDSCs [10, 11]. MDSCs inhibit T cells and NK cells through the secretion of reactive oxygen species (ROS), nitric oxide (NO), and arginase-1 (Arg-1). In some cases, they induce regulatory T cells (Tregs), aiding tumor cells in evading the immune system, thereby rendering the tumor resistant to immunotherapy and promoting tumor progression [6]. Research has elucidated that ascites-derived IL-6 and IL-10 amplify CD14^+^ HLA-DR^−/low^ MDSCs in ovarian cancer patients [12]. Additionally, Snail promotes ovarian cancer progression by recruiting MDSCs through the upregulation of CXCR2 ligands [13]. In the metastasis of colorectal cancer, CXCL1 recruits CXCR2^+^ MDSCs to facilitate premetastatic foci formation [14]. Furthermore, in anti-tumor therapy involving CSF1R blockade, tumor-associated fibroblasts recruit PMN-MDSCs, thereby diminishing therapeutic efficacy [15]. While these studies underscore the pivotal role of MDSCs in tumor development, further investigation is essential to elucidate the key tumor-associated cytokines responsible for the expansion, recruitment, and activation of MDSCs in ovarian cancer and their underlying mechanisms.

Serum amyloid A (SAA) is the major acute phase protein in humans, comprising SAA1, SAA2, SAA3, and SAA4. Synthesized and predominantly secreted by the liver, its levels surge by 1000-fold in response to infections, traumas, cancers, or other inflammatory events [16, 17]. A recent study proposes SAA1 as a potential marker for ovarian cancer metastasis [18]. Furthermore, SAA has been investigated as a potential serum biomarker for various tumors, including lung cancer [19], renal cancer [20], endometrial cancer [21], uterine serous papillary cancer [22], and melanoma [23, 24]. A recent *Nature* study reported an augmented production of SAA during the early stages of pancreatic tumorigenesis in mice. This led to the accumulation of myeloid cells and fibrosis in the liver, promoting the formation of “metastatic ecological niches” and augmenting hepatic metastasis in pancreatic cancer [25]. Nonetheless, no study has elucidated the specific role and underlying mechanisms of SAA1 in ovarian cancer or whether SAA1 can regulate MDSCs to facilitate the development of a tumor-promoting immunosuppressive microenvironment.

In this study, we initially observed a significant accumulation of MDSCs in patients with ovarian cancer, along with a notable upregulation of SAA1. Subsequent in vitro and in vivo experiments demonstrated that SAA1 promotes cancer cell proliferation, migration, and invasion. Co-culture experiments further demonstrated that ovarian cancer cells recruit and activate MDSCs via SAA1 secretion, which engages TLR2/4 on MDSCs and induces IL-1β production. IL-1β, in turn, upregulates SAA1 expression in ovarian cancer cells, establishing a positive feedback loop that promotes the formation of a tumor-promoting immunosuppressive microenvironment and drives ovarian cancer progression. Collectively, these findings elucidate a mechanism by which ovarian cancer cells modulate MDSCs and provide a mechanistic rationale for developing therapeutic strategies targeting ovarian cancer.

## Materials and methods

### Human peripheral blood and tumor tissue samples

Blood and tissue samples were collected from patients with benign gynecological conditions (control group) or epithelial ovarian cancer (EOC group) at Shanghai First Maternity and Infant Hospital between March 2023 and July 2024. The demographic and clinical features of the control and ovarian cancer groups are detailed in Supplementary Table S1. The ovarian cancer group met the following criteria: (1) histopathological confirmation of epithelial ovarian cancer; (2) no chemotherapy or radiotherapy before surgery; and (3) no prior history of malignancy or autoimmune disease. The control group met the following criteria: (1) total hysterectomy with bilateral salpingo-oophorectomy was performed for benign gynecological indications; (2) histopathological confirmation of the absence of ovarian disease; and (3) no history of malignancy or autoimmune disease.

Peripheral blood was collected from patients preoperatively, and peripheral blood mononuclear cells (PBMCs) were isolated using density gradient centrifugation (Ficoll-Paque Plus; GE Healthcare, Pittsburgh, PA, USA) and immediately used for subsequent experiments. After surgery, tumor tissues from patients were digested with trypsin to generate single-cell suspensions for subsequent experiments. The study received approval from the Ethics Committee of Shanghai First Maternity and Infant Hospital (approval number KS2326), and all patients provided written informed consent.

### Flow cytometry

Following the acquisition of single-cell suspensions, surface molecular markers of immune cells were incubated with the corresponding fluorochrome-conjugated antibodies for 30 min at room temperature. To stain molecular markers within the nucleus, staining of cell surface antigens was initially performed as described earlier. Subsequently, nuclear molecular markers were stained using the Pharmingen™ Transcription Factor Buffer Set (562574; BD Biosciences) following the provided instructions for intranuclear markers. For intracellular cytokine staining, single-cell suspensions were initially incubated with Cell Activation Cocktail (with Brefeldin A) (423303; BioLegend) in a 37 °C incubator for 6 h to induce cytokine accumulation. Subsequently, staining of cell surface antigens was completed as described earlier, followed by the intracellular cytokine staining using the Cytofix/Cytoperm™ Fixation/Permeabilization Kit (554714; BD Biosciences) as per the provided instructions. Subsequently, stained cells were acquired on a flow cytometer (BD, FACSCalibur) and analyzed with FlowJo v10.8.1. Different immune cells express distinct molecular markers. In humans, MDSCs are characterized by CD11b^+^CD33^+^HLA-DR^−/low^, M-MDSCs by CD11b^+^CD33^+^CD14^+^, and PMN-MDSCs by CD11b^+^CD33^+^CD15^+^. CD4 + T cells are identified as CD3^+^CD4^+^, while CD8 + T cells are CD3^+^CD8^+^. Tregs are defined by CD4^+^CD25^+^FOXP3^+^. In mice, M-MDSCs are identified as CD45^+^CD11b^+^ Ly6G^−/low^ Ly6C^high^, G-MDSCs as CD45^+^CD11b^+^ Ly6G^high^ Ly6C^−/low^, and Tregs as CD45^+^CD4^+^CD25^+^FOXP3^+^. CD4 + T cells in mice are CD45^+^CD4^+^, and CD8 + T cells are CD45^+^CD8^+^. Additionally, CD8 + T cells in mice secreting IFN-γ are labeled as CD45^+^CD8^+^IFN-γ^+^, and those secreting GZMB as CD45^+^CD8^+^GZMB^+^. Detailed information on the specific flow cytometry antibodies used in this study is provided in Supplementary Table S2.

### RNA extraction, reverse transcription and quantitative real-time polymerase chain reaction (qRT-PCR)

RNA was extracted from cells and tissues using RNAiso Plus (Takara, Japan) following the manufacturer’s instructions. Subsequently, RNA was reverse-transcribed into cDNA using ABScript III RT Master Mix for PCR reagent (ABclonal, China). The cDNA amplification was performed on the QuantStudio™ real-time PCR System (Design & Analysis Software v1.3.1) using Genious 2X SYBR Green Fast qPCR Mix (Low Rox Premixed) (ABclonal, China). The amplification protocol consisted of an initial step at 95 °C for 3 min, followed by 40 cycles of denaturation at 95 °C for 5 s. GAPDH was chosen as an internal reference, and the expression of each gene was normalized using the 2^−ΔΔCT^ method. Synthesis of all primers was carried out by Tsingke Biotech (Shanghai, China). Details of all primers used in this study are provided in Supplementary Table S3.

### Western blotting

Total proteins were extracted from cells and tissues using RIPA lysis buffer (Beyotime, China) supplemented with protease/phosphatase inhibitors and PMSF. Following the preparation of PAGE gels (Epizyme, China) at various concentrations based on protein molecular weights, separation was achieved through 10% SDS-PAGE. After electrophoresis, the proteins were transferred onto a PVDF membrane (Millipore, USA). The membrane was then blocked with 5% skimmed milk for 1 h at room temperature, followed by an overnight incubation at 4 °C with the corresponding primary antibody. The following day, protein visualization was conducted using enhanced chemiluminescence (Millipore, USA) after a 1-hour incubation with secondary antibodies at room temperature. β-Actin was selected as the internal reference for assessing the expression of other proteins. All antibodies used in this study are listed in Supplementary Table S4.

### ELISA

Peripheral blood was collected from patients, and after centrifugation, the serum was obtained and stored at -80 °C. Cells were cultured in 24-well plates for 48 h. Subsequently, the cell culture medium was replaced with an equal volume of serum-free medium, and the cells were cultured for an additional 48 h. Supernatants were collected and stored at -80 °C. Ultimately, the concentrations of SAA1 in both serum and cell supernatants were determined using the SAA1 ELISA kit (ABclonal, China) following the manufacturer’s instructions.

### Immunohistochemistry (IHC)

Tissues from human samples and C57BL/6 mice were collected, fixed in 4% paraformaldehyde, and subsequently embedded in paraffin. Paraffin-embedded sections were deparaffinized in xylene and rehydrated through graded alcohols. The sections were then treated with 3% hydrogen peroxide to quench endogenous peroxidase activity, followed by antigen retrieval. After antigen retrieval, sections were incubated overnight at 4 °C with the primary antibody. The following day, sections were incubated with the corresponding secondary antibody for 1 h at room temperature. Lastly, the sections were stained with DAB and counterstained with hematoxylin.

### Cell culture

The human normal ovarian epithelial cell line (IOSE), human ovarian cancer cell lines (A2780, SKOV3, ES-2, and HEY), human embryonic kidney cell line (293T), and murine-derived ovarian cancer cell line (ID8) were sourced from the American Type Culture Collection (ATCC, USA). IOSE, A2780, SKOV3, and HEY cell lines were cultured in RPMI-1640 medium (VivaCell, China) supplemented with 10% fetal bovine serum (FBS) (Gibco, USA). In contrast, ES-2, 293T, and ID8 were cultured in DMEM medium (VivaCell, China) with 10% FBS. All cell lines were cultured in a 37 °C incubator with 5% CO_2_.

### Lentiviral transfection to construct stable cell lines

The pLKO.1-shSAA1-Puro plasmid for shRNA-mediated knockdown of human SAA1 was designed and synthesized by Tsingke Biotech (Shanghai, China). The PGMLV-shSAA1-Puro plasmid for knockdown of murine SAA1 was designed and synthesized by Geneseed (Shanghai, China). The sgRNA sequence for CRISPR/Cas9-mediated knockout of murine SAA1 was designed by Zhang Lab and synthesized by Tsingke Biotech (Shanghai, China), and subsequently cloned into the lentiCRISPR V2-sgSAA1-Puro vector. In addition, a full-length cDNA sequence of human SAA1 was constructed by You Bao (Hunan, China) for overexpression in ovarian cancer cells. All RNA sequences used for stable cell line construction are listed in Supplementary Table S5.

The recombinant lentiviral plasmids, including the target gene and the packaging plasmids, were initially transfected into 293T cells using PEI (Servicebio, China), and the cell supernatant containing viral particles was collected after 48 h. Upon reaching 60–70% confluency, the target cells were infected with the viral supernatant using co-transfection reagent Polybrene (Geneseed, China). Puromycin was added to select the transfected cells 48 h later, and the remaining cells were the stably transfected cell lines.

### Cell counting Kit-8 (CCK8) assay

The proliferative capacity of cells was assessed using the CCK8 assay. Initially, 1 × 10^3^ cells were seeded per well in a 96-well plate. Subsequently, the culture medium was removed at 0, 24, 48, 72, and 96 h, and 100 µl of serum-free medium containing 10% CCK8 reagent (New Cell & Molecular Biotech, China) was added. Following a 2-hour incubation at 37 °C, the absorbance was measured at 450 nm using a microplate reader.

### 5-Ethynyl-2’-deoxyuridine (EdU) assay

Once the cell confluence reached 60–70%, EdU reagent was added to the culture medium and cells were incubated for 2 h in the incubator. Following fixation with 4% paraformaldehyde, the cells were counterstained with Hoechst 33,342. The proportion of EdU-positive cells was subsequently determined using a fluorescence microscope. This experiment utilized the Cell-Light EdU DNA cell proliferation kit (RiboBio, China).

### Colony formation assay

Target cells (1 × 10³) were seeded per well in 6-well plates and cultured for 1–2 weeks until visible colonies formed. Cells were then fixed with 4% paraformaldehyde for 15 min and stained with 0.1% crystal violet for 15 min. Finally, colonies were counted manually.

### Transwell assay

The Transwell system was assembled by positioning an 8 μm pore-size insert (Corning, USA) atop a 24-well plate. 500 µl of medium containing 20% FBS was added to the lower chamber, and 200 µl of cell suspension in serum-free medium was added to the upper chamber. For invasion experiments, the upper surface of the inserts must be pre-coated with Matrigel (BD Biosciences, USA).

Following 24 h of incubation in a 37 °C incubator, non-migrated cells on the upper surface were removed using a cotton swab. Subsequently, the cells on the lower surface of the inserts were fixed with 4% paraformaldehyde, stained with crystal violet, visualized under a microscope, and quantified with ImageJ software.

### Isolation of MDSCs

Peripheral blood from ovarian cancer patients and single-cell suspensions from the spleens of tumor-bearing mice were isolated by density gradient centrifugation using Ficoll-Paque Plus. The mononuclear cell layer was then aspirated. Subsequently, human CD33 MicroBeads Kit (130-045-501, Miltenyi Biotec) and Mouse CD115 MicroBeads Kit (130-096-354, Miltenyi Biotec) were utilized to isolate MDSCs from human peripheral blood and mouse spleens, respectively.

### MDSCs migration assay

An 8 μm pore size Transwell insert (Corning, USA) was placed in a 24-well plate. In the lower chamber, 500 µl of medium containing 20% FBS or cell supernatants from different treatment groups was added, while 200 µl of MDSCs resuspended in serum-free medium was seeded into the upper chamber. The recombinant protein SAA1 (HY-P70510, human; HY-P700309, mouse; MedChemExpress) was used at a final concentration of 200 ng/ml, and the TLR2/4 inhibitor Sparstolonin B (SsnB; HY-116213, MedChemExpress) was used at a final concentration of 20 µM. Following incubation in a 37 °C incubator for 20–24 h, the MDSCs in the lower chamber were visualized and quantified under a microscope.

### Granulocyte-monocyte progenitors (GMPs) differentiation assay

Umbilical cord blood was collected from healthy donors, and the mononuclear cell layer was extracted following density gradient centrifugation using Ficoll-Paque Plus. Isolated GMPs were cultured with ovarian cancer cell supernatants from various treatment groups. Additionally, 40 ng/mL of IL-6 and 40 ng/mL of GM-CSF were added to the culture system. The concentrations of the recombinant proteins SAA1 and SsnB used in this experiment were set at 200 ng/mL and 20 µM, respectively. After 48 h of incubation, cells were collected for flow cytometry analysis of the proportion of CD11b^+^CD33^+^HLA-DR^−/low^ cells.

### Chromatin Immunoprecipitation (ChIP) assay

Cells were cross-linked with 1% formaldehyde at 37 °C for 10 min. After cell lysis, chromatin was sheared into fragments by sonication. Immunoprecipitation was performed using ChIP-grade P65 antibody (Catalog No. A19653, ABclonal) or IgG (Catalog No. AC042, ABclonal) as a control. Subsequently, ChIP-enriched DNA fragments corresponding to the binding sites on the SAA1 promoter were amplified by qPCR. The ChIP primers used in this study are listed in Supplementary Table S6.

### Peritoneal tumor formation experiments in C57BL/6 mice

Animal care and experiments adhered to the guidelines set by the National Institutes of Health (NIH) and were approved by the Animal Care Committee of Tongji University (approval number TJBA05723101). Ten C57BL/6 female mice, approximately 6 weeks old, were procured from JSJ Laboratory Animal Company (Shanghai, China) and maintained in a specific pathogen-free (SPF) environment. Wild-type and SAA1 knockout murine-derived ovarian cancer cells (ID8) were resuspended in 1×PBS and separately injected into the abdominal cavity of five mice each, with each mouse receiving 1 × 10^7^ ovarian cancer cells.

Periodic observations were made to monitor changes in the abdominal circumference of the mice. After four weeks, an obvious enlargement of the abdominal cavity was observed in wild-type mice. The abdominal circumference of both groups was measured using a flexible ruler. Subsequently, the mice were euthanized, and peritoneal tumor nodules were photographed. Cells were isolated from ascites and spleens for flow cytometry analysis to determine differences in the numbers of different immune cell subsets.

To establish an in vivo imaging model, luciferase-expressing plasmids were stably transfected into both wild-type and SAA1 knockout ID8 cells. The luciferase-labeled ID8 cells were then intraperitoneally injected into female C57BL/6 mice (5 × 10^6^ cells/mouse). Tumor progression was evaluated by quantifying abdominal tumor burden using an in vivo imaging system (IVIS). The endpoint survival time for each mouse was recorded, and at the endpoint, ascitic fluid was aspirated from the peritoneal cavity using a syringe. Abdominal wall tumors were subsequently harvested for immunohistochemical analysis.

### Bioinformatics analysis

Differentially expressed genes in ovarian cancer cells subsequent to co-culture with MDSCs were examined using the GSE145374 dataset from the Gene Expression Omnibus (GEO) database (https://www.ncbi.nlm.nih.gov/geo/). The Gene Expression Profiling Interactive Analysis (GEPIA) database (http://gepia.cancer-pku.cn/) facilitated the assessment of gene expression differences between ovarian cancer tissues and control tissues. The Kaplan–Meier database (https://kmplot.com/analysis/) was employed to evaluate the association between gene expression and survival in ovarian cancer patients. The association between SAA1 and the immune microenvironment in ovarian cancer patients, as well as the correlations among distinct immune cell molecular markers, was investigated based on data from The Cancer Genome Atlas (TCGA) database (https://portal.gdc.cancer.gov/) and the Tumor Immune Estimation Resource (TIMER) database (TIMER2.0 (cistrome.org)). To predict the transcription factors binding to the SAA1 promoter, we utilized three online databases: Joint Annotation of Sequence motifs and Protein Regulatory elements (JASPAR) database (JASPAR - A database of transcription factor binding profiles (elixir.no)), Gene Transcription Regulation Database (GTRD) database (GTRD (biouml.org)), and Human Transcription Factor Database (Human TFDB) database (HumanTFDB (hust.edu.cn)). R software (version 4.1.1) was used for bioinformatics data analysis.

### Statistical analysis

For the classification of patients into high- and low-expression groups for SAA1 and IL1B, the median mRNA expression level (determined by qRT-PCR) within the respective cohort was used as the cutoff value. Patients with expression levels above the median were assigned to the high-expression group, and those at or below the median were assigned to the low-expression group.

Data analysis was performed using GraphPad Prism 9.0.0 (GraphPad Software, USA). Prior to statistical testing, data from human cohorts were examined for normality using the Shapiro–Wilk test. For datasets not normally distributed or with small sample sizes, non-parametric tests (Mann–Whitney U test for two groups, Kruskal–Wallis test for multiple groups) were applied. For normally distributed data, comparisons between two groups were conducted using unpaired two-tailed Student’s t-tests, and multi-group comparisons were performed using one-way ANOVA followed by appropriate post hoc tests (Dunnett’s or Tukey’s). Paired two-tailed Student’s t-tests were applied for matched tumor–adjacent tissue analyses. Survival data were analyzed using the log-rank test, and correlations were assessed using Spearman’s method. Categorical variables were evaluated using the Chi-square (χ²) test. For exploratory mechanistic assays and animal experiments with small sample sizes, consistency across independent biological replicates (in vitro *n* ≥ 3, animal studies *n* = 5 per group) was emphasized, and representative results are presented as mean ± SEM, for which parametric tests such as unpaired Student’s t-tests were applied. A *P* value ≤ 0.05 was considered statistically significant for all analyses.

## Results

### Increased MDSCs and SAA1 expression associated with ovarian cancer

**Fig. 1:**
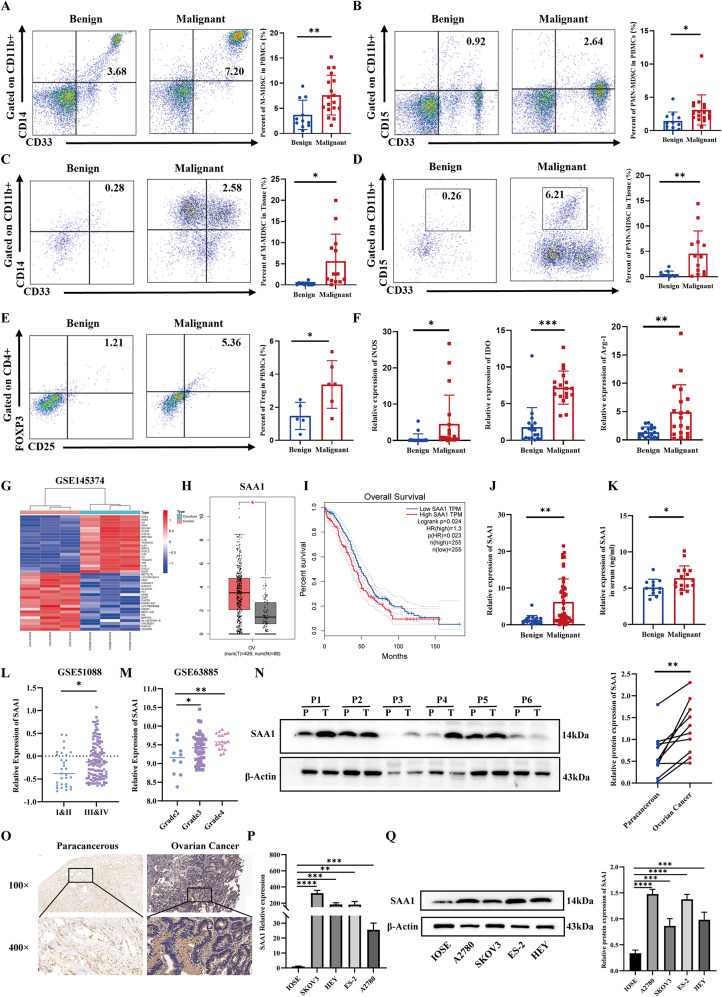
Increased MDSCs and SAA1 expression associated with ovarian cancer. (**A**) Flow cytometry analysis of M-MDSCs in PBMCs from patients with benign and malignant ovarian tumors. Each dot represents an individual patient; data are presented as mean ± SEM; Mann–Whitney U test. (**B**) Flow cytometry analysis of PMN-MDSCs in PBMCs from patients with benign or malignant ovarian tumors. Each dot represents an individual patient; data are presented as mean ± SEM; Mann–Whitney U test. (**C**) Flow cytometry analysis of M-MDSCs in tumor-infiltrating immune cells from patients with benign or malignant ovarian tumors. Each dot represents an individual patient; data are presented as mean ± SEM; Mann–Whitney U test. (**D**) Flow cytometry analysis of PMN-MDSCs in tumor-infiltrating immune cells from patients with benign or malignant ovarian tumors. Each dot represents an individual patient; data are presented as mean ± SEM; Mann–Whitney U test. (**E**) Flow cytometry analysis of Treg cells in PBMCs from patients with benign or malignant ovarian tumors. Each dot represents an individual patient; data are presented as mean ± SEM; Mann–Whitney U test. (**F**) qRT-PCR analysis of the mRNA expression levels of iNOS, IDO and Arg-1 in tumor tissues from patients with benign or malignant ovarian tumors. Each dot represents an individual patient; data are presented as mean ± SEM; Mann–Whitney U test. (**G**) Heatmap showing the top upregulated differentially expressed genes following co-culture of SKOV3 cells and MDSCs based on analysis of the GSE145374 dataset. (**H**) SAA1 expression in ovarian cancer tissues based on the GEPIA database. Each dot represents one sample; data are presented as mean ± SEM; unpaired two-tailed Student’s t-test. (**I**) Kaplan–Meier analysis of overall survival in ovarian cancer patients stratified by SAA1 expression using GEPIA. Log-rank test. (**J**) Detection of SAA1 mRNA expression in benign and malignant ovarian tumor tissues by qRT-PCR. Each dot represents an individual patient; data are presented as mean ± SEM; Mann–Whitney U test. (**K**) Detection of SAA1 protein levels in serum from patients with benign and malignant ovarian tumors by ELISA. Each dot represents an individual patient; data are presented as mean ± SEM; Mann–Whitney U test. (**L**) Association between SAA1 expression and FIGO stage in ovarian cancer based on the public GSE51088 dataset. Each dot represents one sample; Mann–Whitney U test. (**M**) Association between SAA1 expression and histological grade in ovarian cancer based on the public GSE63885 dataset. Each dot represents one sample; Kruskal–Wallis test. (**N**) SAA1 protein expression in paired tumor (T) and adjacent paracancerous (P) tissues assessed by Western blotting (left) and densitometric analysis (right). Each dot represents an individual patient; paired two-tailed Student’s t-test. (**O**) Localization and expression of SAA1 in tumor and paracancerous tissues detected by immunohistochemistry (*n* = 3). Representative images are presented at 100× and 400× magnification. (**P**) SAA1 mRNA expression in normal ovarian epithelial cells and ovarian cancer cell lines assessed by qRT-PCR. Data are presented as mean ± SEM from three independent experiments; one-way ANOVA followed by Dunnett’s multiple comparisons test. (**Q**) Detection of SAA1 protein expression in normal ovarian epithelial cells and ovarian cancer cell lines by Western blotting (left) and densitometric analysis (right). Data are presented as mean ± SEM from three independent experiments; one-way ANOVA followed by Dunnett’s multiple comparisons test. *Statistical significance: *P < 0.05; **P < 0.01; ***P < 0.001; ****P < 0.0001

To explore the role of MDSCs in EOC, we assessed the frequencies of M-MDSCs (CD11b⁺CD33⁺CD14⁺) and PMN-MDSCs (CD11b⁺CD33⁺CD15⁺) in peripheral blood and tumor tissues from patients with benign and malignant ovarian tumors. Flow cytometry revealed significantly higher levels of both M-MDSCs and PMN-MDSCs in EOC patients compared to benign controls (Fig. 1A–D). No significant differences were observed in CD4⁺ or CD8⁺ T cells (Fig. S1A–B), whereas Treg cells were increased in the peripheral blood of EOC patients (Fig. 1E). Detailed immune cell distributions are provided in Supplementary Table S7. Moreover, elevated expression of immunosuppressive molecules iNOS, IDO, and Arg-1 was observed in EOC tissues, with Arg-1 levels being correlated with clinical stage (Fig. 1F; Fig. S1C–E). The MDSC marker S100A8 was also significantly upregulated (Fig. S1F), suggesting MDSC enrichment and enhanced immunosuppressive activity in EOC.

To examine MDSC-tumor interactions, we analyzed the GSE145374 dataset, identifying CSF2, SAA2, IL6, SAA1, and BCL2A1 as the top five upregulated differentially expressed genes in SKOV3 cells after co-culture with MDSCs (Fig. 1G). GEPIA analysis confirmed increased SAA1, SAA2, and BCL2A1 in EOC tissues, with only SAA1 associated with poor overall survival (Fig. 1H–I; Fig. S2A–F). Further exploration in the Kaplan Meier database indicated that elevated SAA1 expression also correlated with shorter Recurrence-Free Survival (RFS) and Post-Progression Survival (PPS) in ovarian cancer patients (Fig. S2G-H).

SAA1 expression was further validated in clinical samples, with significantly elevated mRNA levels observed in EOC tissues (Fig. 1J), and increased serum protein concentrations detected by ELISA in EOC patients compared to benign controls (Fig. 1K). Consistently, analysis of public databases revealed a significant association between high SAA1 expression and both advanced FIGO stage and higher histological grade in ovarian cancer patients (Fig. 1L-M). Moreover, our clinical data further confirmed the positive association between SAA1 expression and FIGO stage in EOC patients (Table 1). At the protein level, SAA1 expression was also increased in tumor tissues relative to adjacent non-tumor tissues (Fig. 1N; Fig. S2I), with immunohistochemistry showing predominant localization in the intercellular space (Fig. 1O). Furthermore, SAA1 was markedly upregulated in ovarian cancer cell lines (SKOV3, HEY, ES-2, A2780) compared to the normal ovarian epithelial cell line IOSE, at both mRNA and protein levels (Fig. 1P–Q).

**Table 1:** Correlation between SAA1/IL1B expression and clinicopathological features in ovarian cancer patients

Characteristics	Cases (49)	SAA1 expression			IL1B expression		
		Low (25)	High (24)	*P*	Low (25)	High (24)	*P*
**Age(years)**				0.316			0.477
≥ 55	25	11	14		14	11	
<55	24	14	10		11	13	
**FIGO stage**				0.005*			0.104
I + II	20	15	5		13	7	
III + IV	29	10	19		12	17	
**Grade**				0.062			0.015*
G1 + G2	23	15	8		16	7	
G3	26	10	16		9	17	
**Peritoneal metastasis**				0.321			0.469
No	23	10	13		13	10	
Yes	26	15	11		12	14	
**CA125(U/mL)**				0.444			0.858
≥ 200	19	11	8		10	9	
<200	30	14	16		15	15	

FIGO International Federation of Gynecology and Obstetrics; *P < 0.05

Two-tailed Chi-square test

Collectively, these results suggest that increased MDSCs and SAA1 expression may contribute to the immunosuppressive features of the EOC microenvironment.

### SAA1 promotes the proliferation of ovarian cancer cells in vitro

**Fig. 2:**
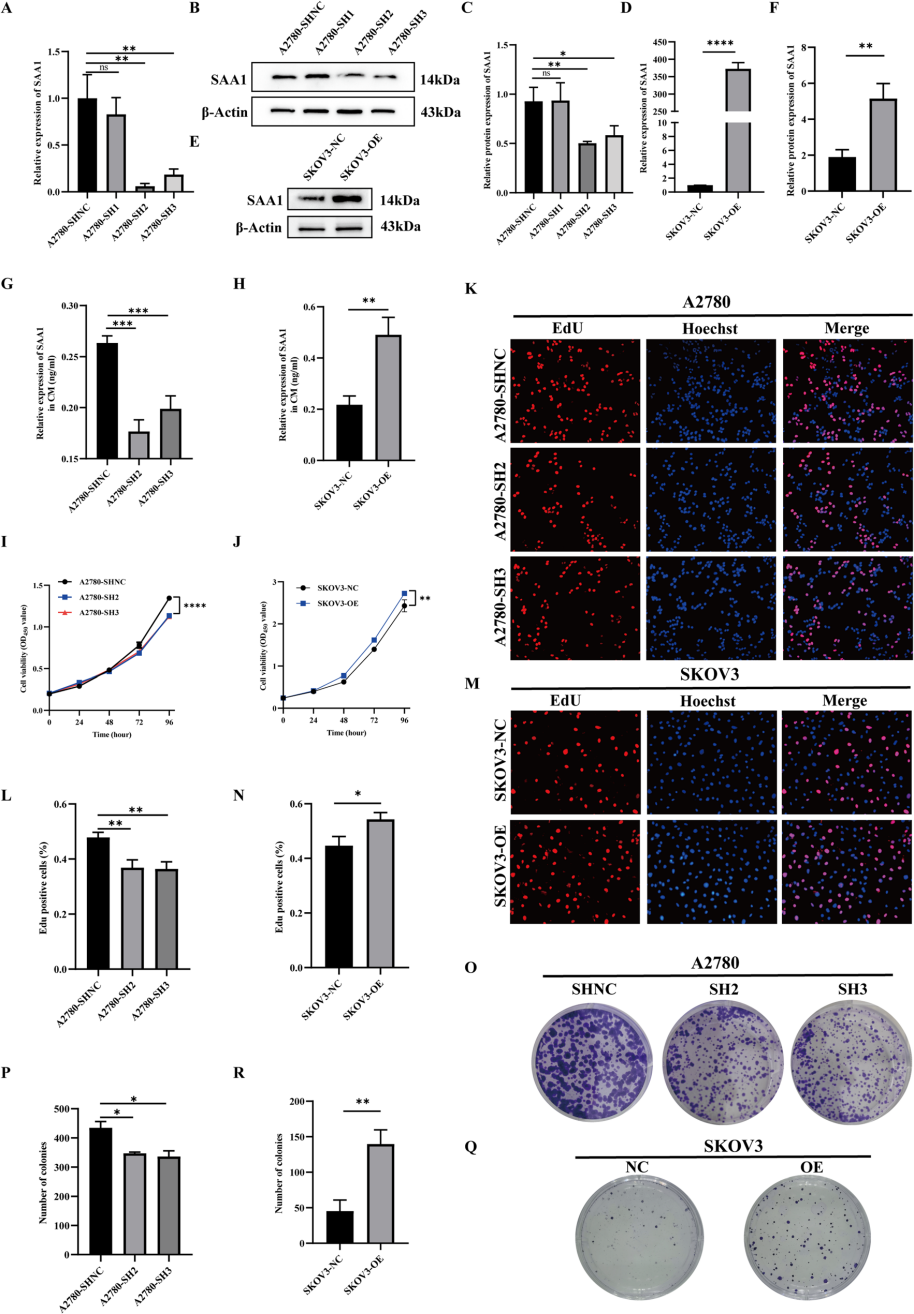
SAA1 promotes the proliferation of ovarian cancer cells in vitro. (**A**) Detection of SAA1 knockdown efficiency at the mRNA level in A2780 cells by qRT-PCR. Data are presented as mean ± SEM from three independent experiments; one-way ANOVA followed by Dunnett’s multiple comparisons test. (**B–C**) Detection of SAA1 knockdown efficiency at the protein level in A2780 cells by Western blotting (**B**) and densitometric analysis (**C**). Data are presented as mean ± SEM from three independent experiments; one-way ANOVA followed by Dunnett’s multiple comparisons test. (**D**) Detection of SAA1 overexpression efficiency at the mRNA level in SKOV3 cells by qRT-PCR. Data are presented as mean ± SEM from three independent experiments; unpaired two-tailed Student’s t-test. (**E–F**) Detection of SAA1 overexpression efficiency at the protein level in SKOV3 cells by Western blotting (**E**) and densitometric analysis (**F**). Data are presented as mean ± SEM from three independent experiments; unpaired two-tailed Student’s t-test. (**G**) Detection of secreted SAA1 levels in the supernatant of A2780 cells after SAA1 knockdown by ELISA. Data are presented as mean ± SEM from three independent experiments; one-way ANOVA followed by Dunnett’s multiple comparisons test. (**H**) Detection of secreted SAA1 levels in the supernatant of SKOV3 cells after SAA1 overexpression by ELISA. Data are presented as mean ± SEM from three independent experiments; unpaired two-tailed Student’s t-test. (**I**) CCK8 assay to evaluate the effect of SAA1 knockdown on A2780 cell proliferation. Data are presented as mean ± SEM from three independent experiments; one-way ANOVA followed by Dunnett’s multiple comparisons test. (**J**) CCK8 assay to evaluate the effect of SAA1 overexpression on SKOV3 cell proliferation. Data are presented as mean ± SEM from three independent experiments; unpaired two-tailed Student’s t-test. (**K–L**) EdU assay to evaluate the proliferative capacity of A2780 cells following SAA1 knockdown. Data are presented as mean ± SEM from three independent experiments; one-way ANOVA followed by Dunnett’s multiple comparisons test. (**M–N**) EdU assay to evaluate the proliferative capacity of SKOV3 cells following SAA1 overexpression. Data are presented as mean ± SEM from three independent experiments; unpaired two-tailed Student’s t-test. (**O–P**) Colony formation assay to assess the clonogenic capacity of A2780 cells following SAA1 knockdown. Data are presented as mean ± SEM from three independent experiments; one-way ANOVA followed by Dunnett’s multiple comparisons test. (**Q–R**) Colony formation assay to assess the clonogenic capacity of SKOV3 cells following SAA1 overexpression. Data are presented as mean ± SEM from three independent experiments; unpaired two-tailed Student’s t-test. *Statistical significance: *P < 0.05; **P < 0.01; ***P < 0.001; ****P < 0.0001; ns, not significant

To delve deeper into the biological role of SAA1 in ovarian cancer cells, we designed three short hairpin RNAs (shRNAs) targeting SAA1 and established a stable SAA1-knockdown A2780 cell line via lentiviral transfection. In parallel, an SKOV3 cell line stably overexpressing SAA1 was generated for subsequent functional analyses. First, the efficiency of SAA1 knockdown in A2780 cells and overexpression in SKOV3 cells was validated by qRT-PCR and Western blotting (Fig. 2A-F). Meanwhile, as SAA1 was knocked down or overexpressed in ovarian cancer cells, the ELISA results further confirmed that SAA1 in the cell supernatants was also decreased or increased with it (Fig. 2G-H). Subsequent analyses using CCK-8 assays and EdU assays revealed that SAA1 knockdown significantly inhibited the proliferation of ovarian cancer cells, while overexpression of SAA1 markedly promoted their proliferation (Fig. 2I-N). Moreover, clonogenic assays were employed to assess the impact of SAA1 on the clonogenic capacity of ovarian cancer cells, demonstrating that SAA1 knockdown led to a substantial decrease in clonogenicity, whereas SAA1 overexpression significantly enhanced this capacity (Fig. 2O-R). Furthermore, a stable murine ovarian cancer cell line (ID8) with SAA1 knockdown was established (Fig. S3A-B). Consistent with our findings in human ovarian cancer cells, CCK-8 assays, EdU assays, and clonogenic assays in ID8 cells corroborated that SAA1 knockdown significantly inhibited proliferation and clonogenic capacity (Fig. S3C-G). In summary, these results strongly suggest that SAA1 promotes the proliferation and clonogenic capacity of ovarian cancer cells in vitro.

### SAA1 promotes the proliferation and metastasis of ovarian cancer in vitro and in vivo

**Fig. 3:**
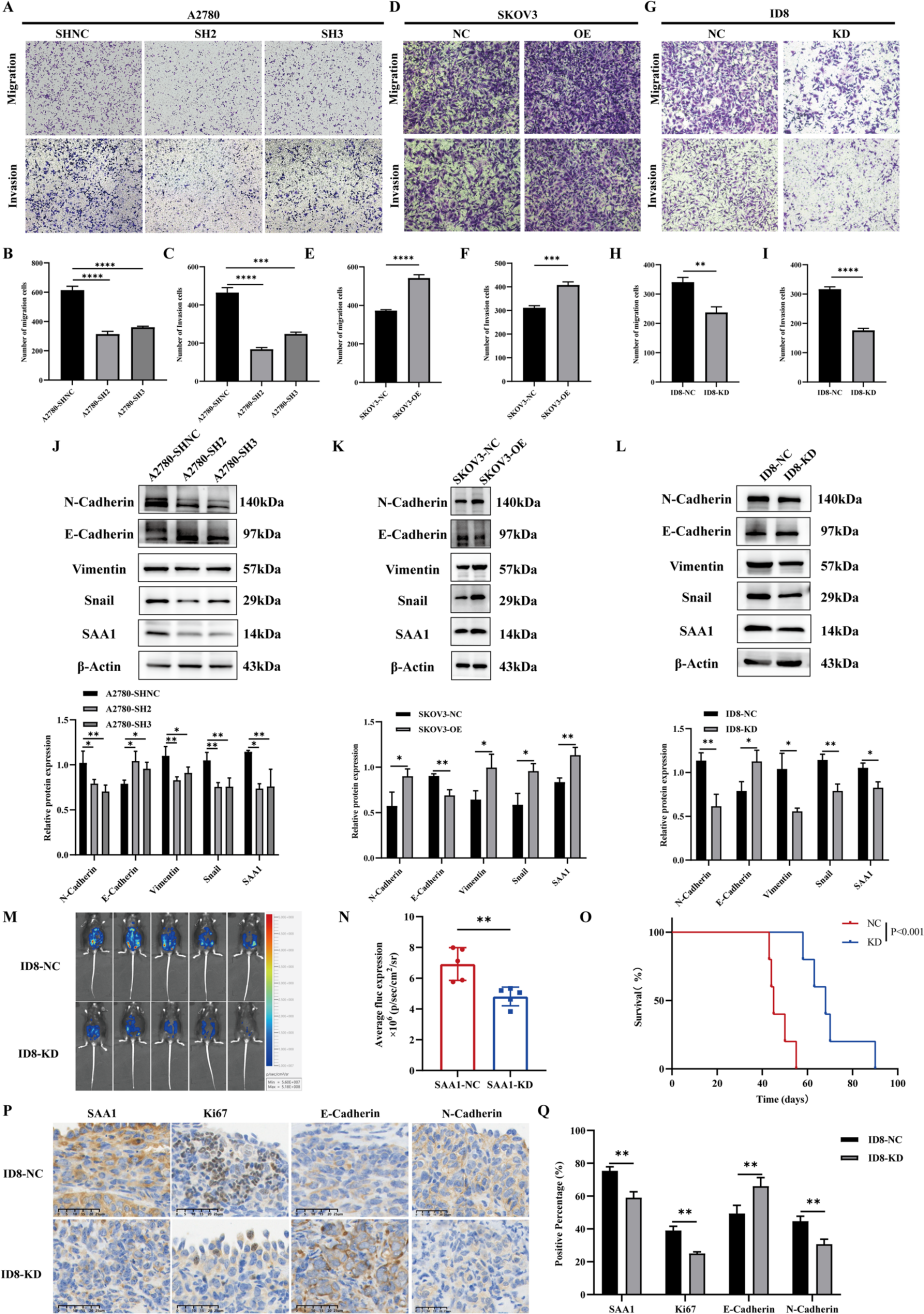
SAA1 promotes the proliferation and metastasis of ovarian cancer in vivo and in vitro. (**A–C**) Transwell assay to evaluate the migration and invasion capacity of A2780 cells following SAA1 knockdown. Data are presented as mean ± SEM from three independent experiments; one-way ANOVA followed by Dunnett’s multiple comparisons test. (**D–F**) Transwell assay to evaluate the migration and invasion capacity of SKOV3 cells following SAA1 overexpression. Data are presented as mean ± SEM from three independent experiments; unpaired two-tailed Student’s t-test. (**G–I**) Transwell assay to evaluate the migration and invasion capacity of ID8 cells following SAA1 knockdown. Data are presented as mean ± SEM from three independent experiments; unpaired two-tailed Student’s t-test. (**J**) Detection of EMT-related proteins (N-Cadherin, E-Cadherin, Vimentin, Snail) in A2780 cells following SAA1 knockdown by Western blotting (top) and densitometric analysis (bottom). Data are presented as mean ± SEM from three independent experiments; one-way ANOVA followed by Dunnett’s multiple comparisons test. (**K**) Detection of EMT-related proteins in SKOV3 cells following SAA1 overexpression by Western blotting (top) and densitometric analysis (bottom). Data are presented as mean ± SEM from three independent experiments; unpaired two-tailed Student’s t-tests. (**L**) Detection of EMT-related proteins in ID8 cells following SAA1 knockdown by Western blotting (top) and densitometric analysis (bottom). Data are presented as mean ± SEM from three independent experiments; unpaired two-tailed Student’s t-tests. (**M–N**) Luciferase-based in vivo imaging to evaluate the effect of SAA1 knockdown on ovarian cancer progression in tumor-bearing mice. Each dot represents one mouse; data are presented as mean ± SEM; unpaired two-tailed Student’s t-test. (**O**) Kaplan–Meier analysis to evaluate the effect of SAA1 knockdown on the survival duration of tumor-bearing mice. Log-rank test. (**P–Q**) Immunohistochemical detection of SAA1, Ki67, E-Cadherin, and N-Cadherin expression in abdominal wall tumors of tumor-bearing mice (**P**) and quantification of positive staining (**Q**). Data are presented as mean ± SEM from three mice per group; unpaired two-tailed Student’s t-tests. *Statistical significance: *P < 0.05; **P < 0.01; ***P < 0.001; ****P < 0.0001

Furthermore, we conducted Transwell assays to investigate the potential role of SAA1 in the metastatic process of ovarian cancer cells. The results revealed a significant decrease in the migration and invasion abilities of A2780 cells following SAA1 knockdown (Fig. 3A-C), whereas these abilities were markedly enhanced upon overexpression of SAA1 in SKOV3 cells (Fig. 3D-F). Consistently, the migration and invasion capabilities of ID8 cells were significantly reduced after SAA1 knockdown (Fig. 3G-I). Given that the migration and invasion of cancer cells are closely associated with epithelial-to-mesenchymal transition (EMT), we examined alterations in key molecular proteins within the EMT pathway using Western blotting. The results demonstrated that, following SAA1 knockdown, the expression of the epithelial marker E-Cadherin increased, while the mesenchymal markers N-Cadherin, Vimentin, and Snail were all significantly downregulated. Conversely, upon SAA1 overexpression, epithelial markers were significantly downregulated, and the aforementioned mesenchymal markers were all markedly upregulated (Fig. 3J-L). In short, our findings indicate that SAA1 promotes the metastasis of ovarian cancer cells through the EMT pathway in vitro.

To further investigate the role of SAA1 in ovarian cancer progression, we transfected a luciferase plasmid into SAA1-knockdown murine ovarian cancer cell line ID8 for in vivo imaging. Wild-type and SAA1-knockdown ID8 cells, both expressing luciferase, were intraperitoneally injected into C57BL/6 mice. At the survival endpoint, the knockdown group showed significantly lower fluorescence intensity and prolonged survival compared to the wild-type group (Fig. 3M-O). Immunohistochemical analysis of tumor tissues revealed a marked reduction in SAA1 expression in the knockdown group, along with decreased Ki67 and N-Cadherin levels and increased E-Cadherin expression (Fig. 3P-Q). These results demonstrate that SAA1 promotes ovarian cancer progression both in vitro and in vivo.

### SAA1 recruits MDSCs and promotes MDSCs differentiation in ovarian cancer

**Fig. 4:**
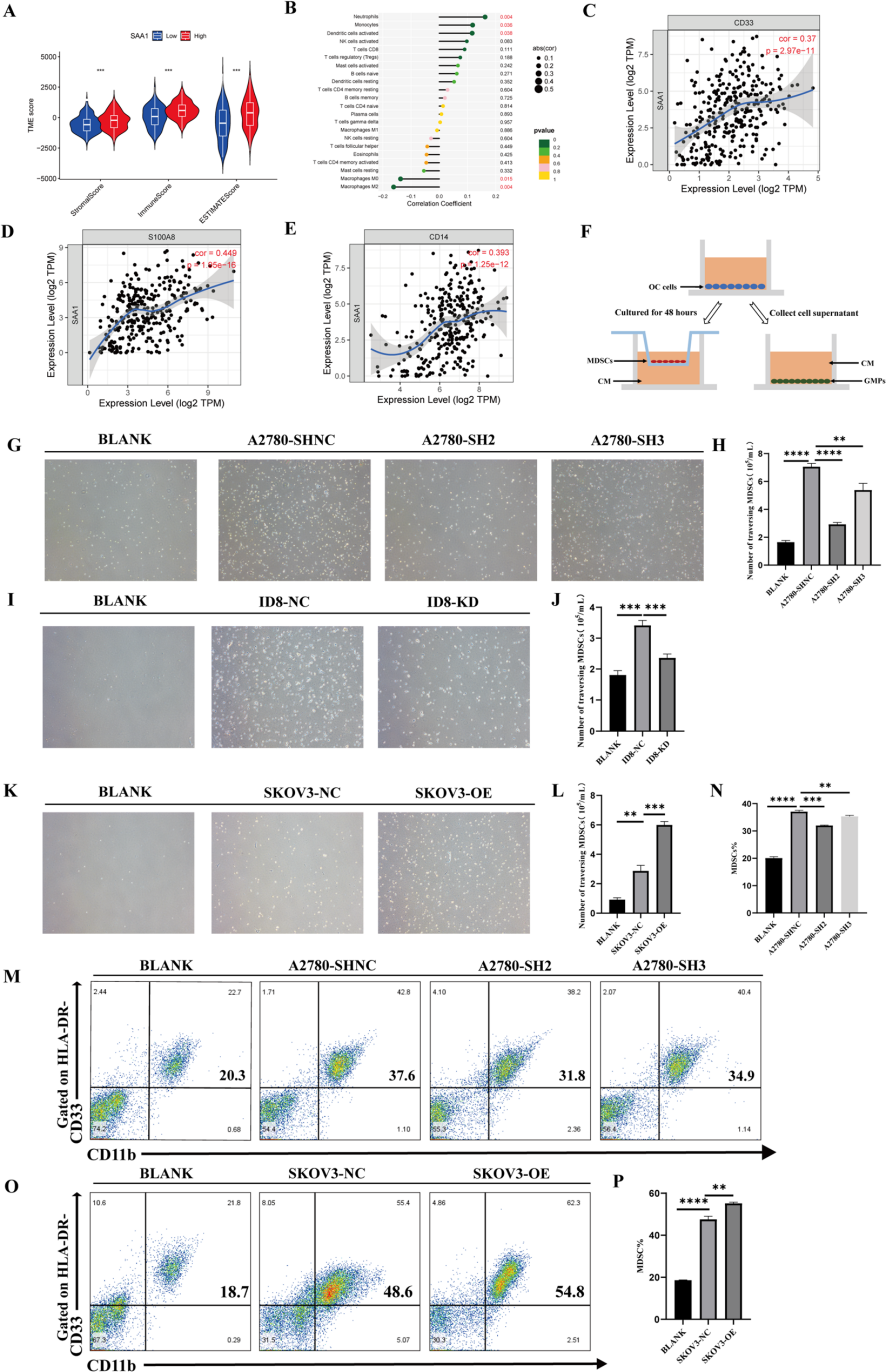
SAA1 recruits MDSCs and promotes MDSCs differentiation in ovarian cancer. (**A**) Relationship between SAA1 expression and immune infiltration in ovarian cancer analyzed using the CIBERSORT algorithm based on TCGA data. (**B**) Correlation between SAA1 expression and the infiltration of different immune cell subsets in ovarian cancer using the CIBERSORT algorithm based on TCGA data. (**C–E**) Correlation analysis between SAA1 expression and CD33 (**C**), S100A8 (**D**), or CD14 (**E**) expression in ovarian cancer using the TIMER database; Spearman’s rank correlation test. (**F**) Schematic diagram of MDSCs recruitment and pro-GMP differentiation experiments. (**G–H**) Co-culture assays to detect the ability of supernatants from A2780 cells with SAA1 knockdown to recruit MDSCs. Data are presented as mean ± SEM from three independent experiments; one-way ANOVA followed by Dunnett’s multiple comparisons test. (**I–J**) Co-culture assays to detect the ability of supernatants from ID8 cells with SAA1 knockdown to recruit MDSCs. Data are presented as mean ± SEM from three independent experiments; one-way ANOVA followed by Dunnett’s multiple comparisons test. (**K–L**) Co-culture assays to detect the ability of supernatants from SKOV3 cells with SAA1 overexpression to recruit MDSCs. Data are presented as mean ± SEM from three independent experiments; one-way ANOVA followed by Dunnett’s multiple comparisons test. (**M–N**) Flow cytometry to detect the ability of supernatants from A2780 cells with SAA1 knockdown to promote the differentiation of GMPs to MDSCs. Data are presented as mean ± SEM from three independent experiments; one-way ANOVA followed by Dunnett’s multiple comparisons test. (**O–P**) Flow cytometry to detect the ability of supernatants from SKOV3 cells with SAA1 overexpression to promote the differentiation of GMPs to MDSCs. Data are presented as mean ± SEM from three independent experiments; one-way ANOVA followed by Dunnett’s multiple comparisons test. *Statistical significance: **P < 0.01; ***P < 0.001; ****P < 0.0001

Previous experiments have provided initial evidence of the association between SAA1 and MDSCs. However, a more in-depth exploration of the role that SAA1 plays in the immune microenvironment of ovarian cancer is warranted. Utilizing the TCGA database, we scored the immune microenvironment of ovarian cancer based on the expression levels of SAA1. The results revealed a higher immune infiltration score in the SAA1 high expression group (Fig. 4A). Further analysis demonstrated a significant positive correlation between SAA1 and neutrophils, monocytes, as well as with MDSC subtypes—granulocyte MDSCs (PMN-MDSCs) and monocyte MDSCs (M-MDSCs) (Fig. 4B). Additionally, SAA1 showed a significant positive correlation with key molecular markers CD33 and S100A8 for MDSCs, and with the marker CD14 for M-MDSCs (Fig. 4C-E). These bioinformatics analysis results provide initial insights into the significant correlation between SAA1 and MDSCs, underscoring its pivotal role in the immune microenvironment of ovarian cancer.

To investigate whether SAA1 released by ovarian cancer cells could recruit MDSCs, we collected cell supernatants from ovarian cancer cells and placed them in the lower chamber of a co-culture system, and MDSCs in the upper chamber. After 24 h, we assessed the number of MDSCs migrating to the lower chamber (Fig. 4F). Results demonstrated that ovarian cancer cell supernatants significantly recruited MDSCs compared to the blank group. This recruitment ability was notably reduced after knocking down SAA1 in ovarian cancer cells A2780 and ID8 (Fig. 4G-J). Conversely, overexpression of SAA1 in ovarian cancer cells SKOV3 led to a substantial enhancement in the recruitment of MDSCs by their supernatants (Fig. 4K-L). Furthermore, we cultured granulocyte-monocyte progenitors (GMPs) with supernatants from different groups of cancer cell lines. The results indicated that supernatants from ovarian cancer cells significantly contributed to the differentiation of GMPs toward MDSCs. Specifically, supernatants from A2780 cells with SAA1 knockdown exhibited a significant decrease in their pro-differentiation capacity (Fig. 4M-N), while those from SKOV3 cells overexpressing SAA1 induced a significant increase in their pro-differentiation capacity (Fig. 4O-P). In conclusion, these findings establish the crucial role of SAA1 in the recruitment and differentiation of MDSCs in ovarian cancer.

### SAA1 released by ovarian cancer cells recruits MDSCs and promotes MDSCs differentiation via TLR2/4

**Fig. 5:**
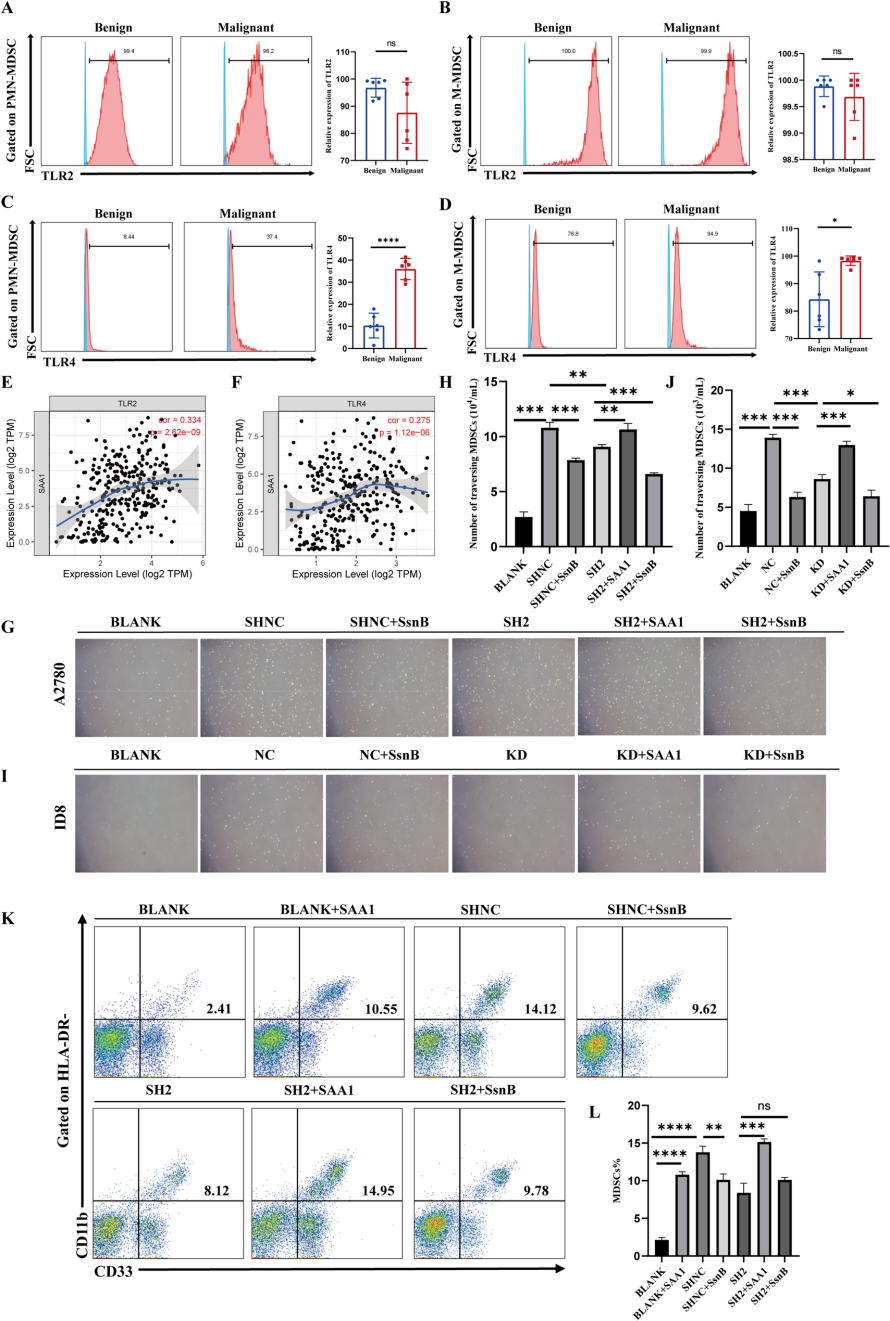
SAA1 released by ovarian cancer cells recruits MDSCs and promotes MDSCs differentiation via TLR2/4. (**A**–**B**) Flow cytometry analysis of TLR2 expression on the surface of PMN-MDSCs (**A**) and M-MDSCs (**B**) from the peripheral blood of patients with benign or malignant ovarian tumors. Each dot represents an individual patient; data are presented as mean ± SEM; Mann–Whitney U test. (**C–D**) Flow cytometry analysis of TLR4 expression on the surface of PMN-MDSCs (**C**) and M-MDSCs (**D**) from the peripheral blood of patients with benign or malignant ovarian tumors. Each dot represents an individual patient; data are presented as mean ± SEM; Mann–Whitney U test. (**E–F**) Correlation analysis between SAA1 expression and TLR2 (**E**) or TLR4 (**F**) expression in ovarian cancer using the TIMER database; Spearman’s rank correlation test. (**G–H**) Rescue experiments assessing the effect of adding recombinant SAA1 protein or the TLR2/4 inhibitor SsnB on the ability of A2780 cell supernatants to recruit MDSCs. Data are presented as mean ± SEM from three independent experiments; one-way ANOVA followed by Tukey’s multiple comparisons test. (**I–J**) Rescue experiments assessing the effect of recombinant SAA1 protein or SsnB on the ability of ID8 cell supernatants to recruit MDSCs. Data are presented as mean ± SEM from three independent experiments; one-way ANOVA followed by Tukey’s multiple comparisons test. (**K–L**) Rescue experiments assessing the effect of adding recombinant SAA1 protein or SsnB on the ability of A2780 cell supernatants to induce GMP differentiation into MDSCs. Data are presented as mean ± SEM from three independent experiments; one-way ANOVA followed by Tukey’s multiple comparisons test. *Statistical significance: *P < 0.05; **P < 0.01; ***P < 0.001; ****P < 0.0001; ns, not significant

SAA1 in ovarian cancer cells has been linked to the recruitment and differentiation of MDSCs, prompting further investigation into the underlying mechanisms. Existing research indicates that SAA1 primarily interacts with receptors including TLR2, TLR4, and FPR2. Notably, TLR2/4 play a pivotal role in the recruitment and activation of MDSCs. Therefore, we hypothesized that SAA1 released by ovarian cancer cells could recruit MDSCs and facilitate the differentiation of GMPs into MDSCs via TLR2/4 on MDSCs. Flow cytometry was first used to examine the expression of TLR2/4 on MDSCs in peripheral blood of patients with benign or malignant tumors. The results showed no significant difference in TLR2 expression on MDSC subsets, whereas TLR4 was upregulated in malignant ovarian tumors, particularly on PMN-MDSCs (Fig. 5A-D). In addition, correlation analysis using the TIMER database revealed a significant positive association between SAA1 and TLR2/4 (Fig. 5E-F).

In addition, a series of rescue experiments were conducted using recombinant protein SAA1 and Sparstolonin B (SsnB), an inhibitor of TLR2/4. The addition of recombinant protein SAA1 to the supernatants of SAA1 knockdown A2780 cells restored their ability to recruit MDSCs. Conversely, the addition of SsnB to the supernatants of negative control A2780 cells significantly inhibited their ability to recruit MDSCs (Fig. 5G-H). Consistent findings were observed in experiments with ID8 cells (Fig. 5I-J). Moreover, differentiation experiments with GMPs demonstrated that the addition of recombinant SAA1 restored the diminished pro-differentiation capacity of A2780 cell supernatants caused by SAA1 knockdown. Conversely, the addition of SsnB significantly impaired the ability of A2780 cell supernatants to promote the differentiation of GMPs into MDSCs (Fig. 5K-L). In conclusion, ovarian cancer cells release SAA1, which acts on the TLR2/4 on the surface of MDSCs, thereby recruiting MDSCs and promoting the differentiation of GMPs into MDSCs.

### SAA1–mediated IL-1β release from MDSCs enhances SAA1 expression in ovarian cancer via the IL-1β/IL-1R/NF-κB axis

**Fig. 6:**
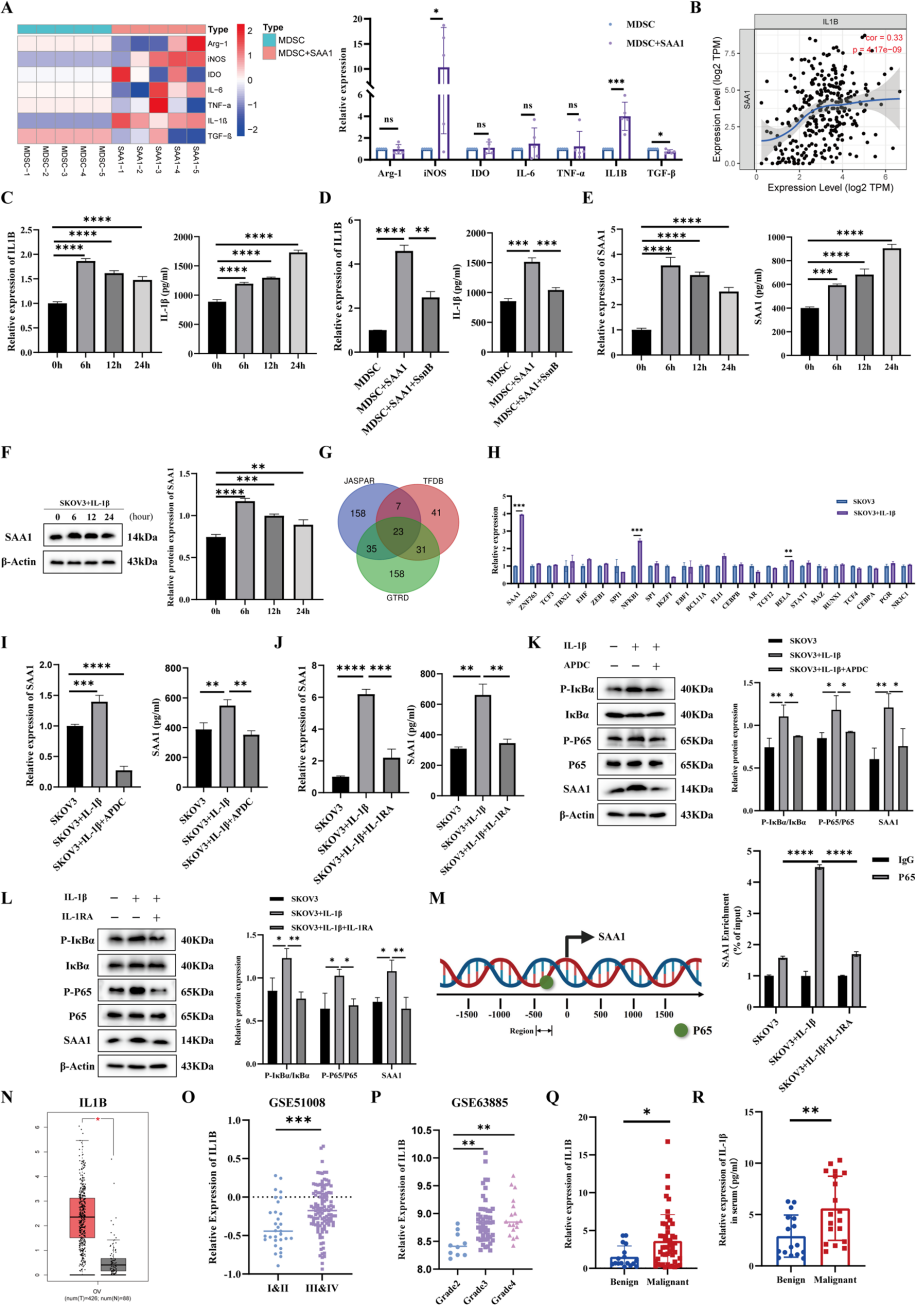
SAA1–mediated IL-1β release from MDSCs enhances SAA1 expression in ovarian cancer via the IL-1β/IL-1R/NF-κB axis. (**A**) Cytokine expression profile in MDSCs following SAA1 stimulation, detected by qRT-PCR. Data are presented as mean ± SEM from five independent experiments; unpaired two-tailed Student’s t-test. (**B**) Correlation analysis between SAA1 and IL1B expression in ovarian cancer using the TIMER database; Spearman’s rank correlation test. (**C**) IL-1β expression and secretion in MDSCs after SAA1 treatment across time points (0, 6, 12, 24 h), detected by qRT-PCR (left) and ELISA (right). Data are presented as mean ± SEM from three independent experiments; one-way ANOVA followed by Dunnett’s multiple comparisons test. (**D**) IL-1β expression and secretion in MDSCs after adding the TLR2/4 inhibitor SsnB to SAA1 treatment, detected by qRT-PCR (left) and ELISA (right). Data are presented as mean ± SEM from three independent experiments; one-way ANOVA followed by Dunnett’s multiple comparisons test. (**E**) SAA1 expression and secretion in SKOV3 cells after IL-1β treatment across time points (0, 6, 12, 24 h), detected by qRT-PCR (left) and ELISA (right). Data are presented as mean ± SEM from three independent experiments; one-way ANOVA followed by Dunnett’s multiple comparisons test. (**F**) Detection of SAA1 protein expression in SKOV3 cells after IL-1β treatment across time points (0, 6, 12, 24 h) by Western blotting (left) and densitometric analysis (right). Data are presented as mean ± SEM from three independent experiments; one-way ANOVA followed by Dunnett’s multiple comparisons test. (**G**) Prediction of transcription factors binding to the SAA1 promoter using TFDB, GTRD, and JASPAR databases. (**H**) Expression of predicted transcription factors in SKOV3 cells with or without IL-1β treatment, detected by qRT-PCR. Data are presented as mean ± SEM from three independent experiments; unpaired two-tailed Student’s t-tests. (**I**) SAA1 expression and secretion in SKOV3 cells after adding the NF-κB inhibitor APDC to IL-1β treatment, detected by qRT-PCR (left) and ELISA (right). Data are presented as mean ± SEM from three independent experiments; one-way ANOVA followed by Dunnett’s multiple comparisons test. (**J**) SAA1 expression and secretion in SKOV3 cells after adding the IL-1 receptor antagonist (IL-1RA) to IL-1β treatment, detected by qRT-PCR (left) and ELISA (right). Data are presented as mean ± SEM from three independent experiments; one-way ANOVA followed by Dunnett’s multiple comparisons test. (**K**) Detection of NF-κB signaling molecules in SKOV3 cells after adding APDC to IL-1β treatment by Western blotting (left) and densitometric analysis (right). Data are presented as mean ± SEM from three independent experiments; one-way ANOVA followed by Dunnett’s multiple comparisons test. (**L**) Detection of NF-κB signaling molecules in SKOV3 cells after adding IL-1RA to IL-1β treatment by Western blotting (left) and densitometric analysis (right). Data are presented as mean ± SEM from three independent experiments; one-way ANOVA followed by Dunnett’s multiple comparisons test. (**M**) ChIP assay to detect the binding of transcription factor P65 to the SAA1 promoter in SKOV3 cells. Data are presented as mean ± SEM from three independent experiments; one-way ANOVA followed by Dunnett’s multiple comparisons test. (**N**) IL1B expression in ovarian cancer tissues based on the GEPIA database. Each dot represents one sample; data are presented as mean ± SEM; unpaired two-tailed Student’s t-test. (**O**) Association between IL1B expression and FIGO stage in ovarian cancer based on the GSE51088 dataset. Each dot represents one sample; Mann–Whitney U test. (**P**) Association between IL1B expression and histological grade in ovarian cancer based on the GSE63885 dataset. Each dot represents one sample; Kruskal–Wallis test. (**Q**) Detection of IL1B mRNA expression in benign and malignant ovarian tumor tissues by qRT-PCR. Each dot represents an individual patient; data are presented as mean ± SEM; Mann–Whitney U test. (**R**) Detection of IL-1β protein levels in serum from patients with benign and malignant ovarian tumors by ELISA. Each dot represents an individual patient; data are presented as mean ± SEM; Mann–Whitney U test. *Statistical significance: *P < 0.05; **P < 0.01; ***P < 0.001; ****P < 0.0001; ns, not significant

Studies have shown that SAA1, as an acute inflammation-related protein, promotes immune cells to secrete cytokines such as IL-1β, IL-6, and TNF-α [26]. Moreover, MDSCs, as immunosuppressive cells, also secrete immunosuppressive factors such as Arg-1, iNOS, and IDO. Therefore, we first explored the effect of SAA1 on the secretion of cytokines by MDSCs. The results showed that SAA1 indeed promoted cytokine secretion by MDSCs, with IL-1β being the most significantly upregulated (Fig. 6A). In addition, analysis revealed a positive correlation between SAA1 and IL1B expression in ovarian cancer tissues (Fig. 6B). Further experiments confirmed that SAA1 enhanced IL-1β expression at both the mRNA and protein levels (Fig. 6C). After adding the TLR2/4 inhibitor SsnB, the ability of SAA1 to promote IL-1β secretion by MDSCs was significantly inhibited, suggesting that SAA1 promotes IL-1β secretion through the TLR2/4 signaling (Fig. 6D).

Considering that IL-1β is also a potent inducer of SAA1 release, we further investigated whether IL-1β secreted by MDSCs could, in turn, stimulate SAA1 production in ovarian cancer cells. The results confirmed that IL-1β significantly upregulated both the expression and secretion of SAA1 in ovarian cancer cells (Fig. 6E-F). To elucidate the underlying regulatory mechanism, we utilized the TFDB, GTRD, and JASPAR databases to predict transcription factors potentially involved in SAA1 regulation. Among the overlapping candidates, NF-κB1 and RELA exhibited significant differential expression at the RNA level, implicating the NF-κB signaling pathway in this process (Fig. 6G-H). The application of the NF-κB pathway inhibitor APDC markedly suppressed IL-1β-induced SAA1 secretion in ovarian cancer cells (Fig. 6I, K). Furthermore, inhibition of the IL-1β receptor (IL-1R) using IL-1RA similarly reduced IL-1β-driven SAA1 secretion (Fig. 6J, L). ChIP assays further confirmed that the transcription factor P65 (RELA) directly binds to the SAA1 promoter, thereby promoting its transcription (Fig. 6M, Fig. S4). Finally, IL1B was found to be significantly upregulated in ovarian cancer tissues based on GEO database analyses and was associated with advanced stage and poor grade (Fig. 6N-P). This was further confirmed by our detection of elevated IL-1β levels in clinical tissue and serum samples, and a similar association with pathological grade was also observed in our patient cohort (Fig. 6Q-R; Table 1).

These findings suggest that SAA1 promotes IL-1β secretion by binding to TLR2/4 on the surface of MDSCs. In turn, IL-1β acts on ovarian cancer cells to enhance SAA1 expression and release via the IL-1β/IL-1R/NF-κB signaling pathway.

### Deciphering the impact of SAA1-driven immunosuppression and its clinical relevance in ovarian cancer

**Fig. 7:**
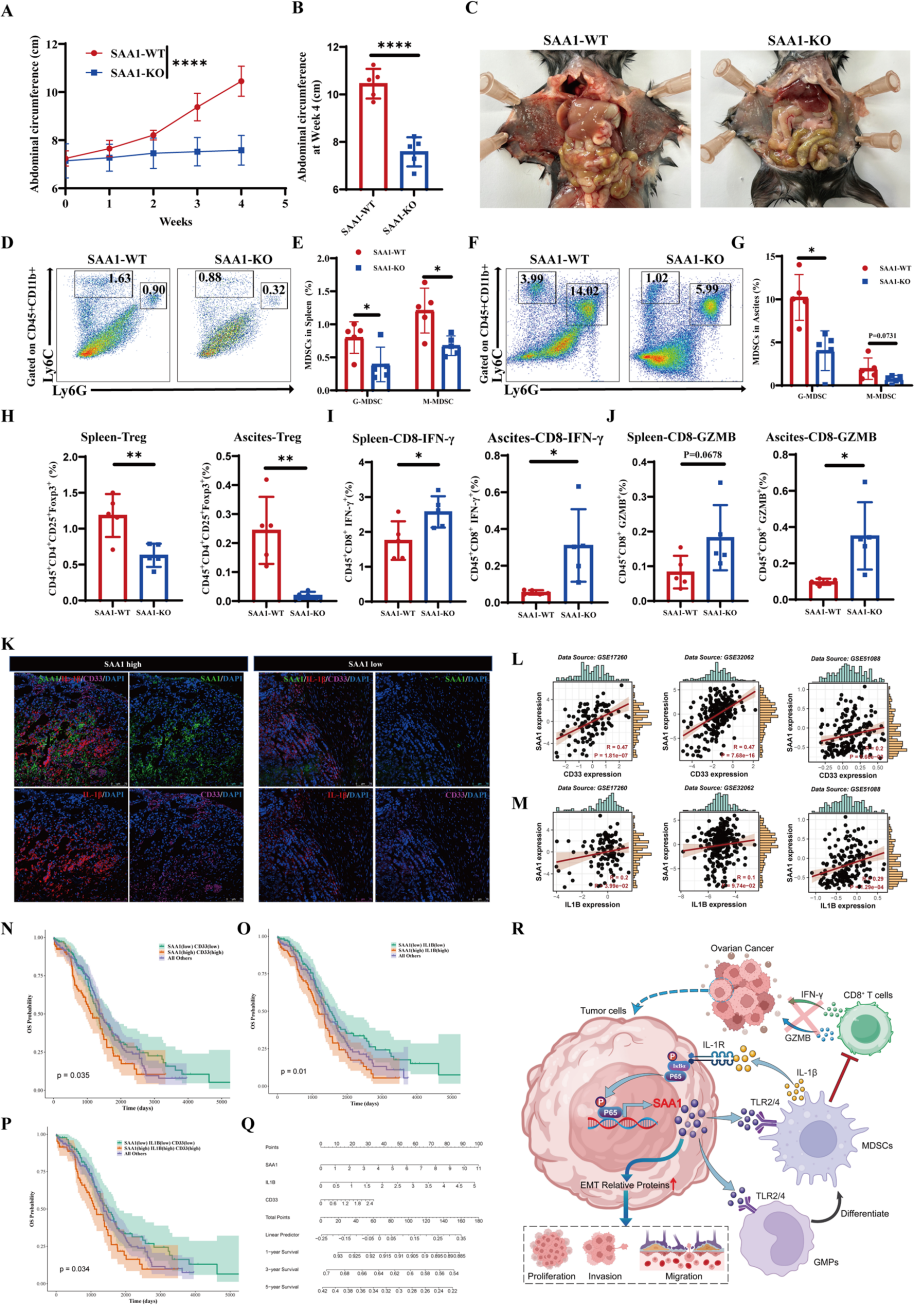
Deciphering the impact of SAA1-driven immunosuppression and its clinical relevance in ovarian cancer. (**A**) Abdominal circumference of C57BL/6 mice measured weekly for 4 weeks after intraperitoneal injection of SAA1-WT or SAA1-KO ID8 cells. Data are presented as mean ± SEM; two-way ANOVA. (**B**) Abdominal circumference at Week 4. Each dot represents one mouse; data are presented as mean ± SEM; unpaired two-tailed Student’s t-test. (**C**) Representative gross images showing differences in tumor nodule formation on the abdominal wall between SAA1-WT and SAA1-KO mice. (**D–E**) Detection of splenic G-MDSCs and M-MDSCs in SAA1-WT and SAA1-KO mice by flow cytometry (**D**) and quantification (**E**). Each dot represents one mouse; data are presented as mean ± SEM; unpaired two-tailed Student’s t-tests. (**F–G**) Detection of ascitic G-MDSCs and M-MDSCs in SAA1-WT and SAA1-KO mice by flow cytometry (**F**) and quantification (**G**). Each dot represents one mouse; data are presented as mean ± SEM; unpaired two-tailed Student’s t-tests. (**H**) Flow cytometry analysis of the number of Treg cells in the spleen and ascites of SAA1-WT and SAA1-KO mice. Each dot represents one mouse; data are presented as mean ± SEM; unpaired two-tailed Student’s t-test. (**I**) Flow cytometry to detect the ability of CD8⁺ T cells to secrete IFN-γ in the spleen and ascites of SAA1-WT and SAA1-KO mice. Each dot represents one mouse; data are presented as mean ± SEM; unpaired two-tailed Student’s t-test. (**J**) Flow cytometry to detect the ability of CD8⁺ T cells to secrete GZMB in the spleen and ascites of SAA1-WT and SAA1-KO mice. Each dot represents one mouse; data are presented as mean ± SEM; unpaired two-tailed Student’s t-test. (**K**) Representative multiplex immunofluorescence (mIF) images of tumor tissues from ovarian cancer patients with high or low SAA1 expression, with three patients in each group. (**L**) Correlation between SAA1 and CD33 expression in the GSE17260, GSE32062, and GSE51088 datasets; Spearman’s rank correlation test. (**M**) Correlation between SAA1 and IL1B expression in the GSE17260, GSE32062, and GSE51088 datasets; Spearman’s rank correlation test. (**N**) Kaplan–Meier analysis evaluating the prognostic value of SAA1^high^CD33^high^ versus SAA1^low^CD33^low^ expression for OS in TCGA-OC patients; log-rank test. (**O**) Kaplan–Meier survival analysis evaluating the prognostic value of SAA1^high^IL1B^high^ versus SAA1^low^IL1B^low^ expression for OS in TCGA-OC patients; log-rank test. (**P**) Kaplan–Meier survival analysis evaluating the prognostic value of the SAA1/IL1B/CD33 axis for OS in TCGA-OC patients; log-rank test. (**Q**) A nomogram constructed based on SAA1, IL1B, and CD33 expression from TCGA-OC data. (**R**) Schematic diagram showing how ovarian cancer cells form a positive feedback loop with MDSCs through the SAA1-IL-1β axis, promoting immune evasion. *Statistical significance: *P < 0.05; **P < 0.01; ****P < 0.0001; ns, not significant

To further elucidate the pivotal role of SAA1 in ovarian carcinogenesis in vivo, we employed CRISPR/Cas9 technology to generate a murine ovarian cancer ID8 cell line with SAA1 knockout (SAA1-KO) (Fig. S5A). These SAA1-KO cells, along with wild-type ID8 cells (SAA1-WT), were injected intraperitoneally into C57BL/6 mice. Abdominal circumference progressively increased in the SAA1-WT group, and was significantly larger than that of the SAA1-KO group at Week 4 (Fig. 7A-B). Upon dissection, fewer tumor nodules were observed on the abdominal wall in the SAA1-KO group (Fig. 7C; Fig. S5C). Additionally, all mice in the SAA1-WT group developed hemorrhagic ascites (Fig. S5B). Flow cytometry was performed on ascites and spleen samples to assess the impact of SAA1 knockout on the immunosuppressive microenvironment. Mice in the SAA1-WT group showed significantly higher levels of MDSCs, including both G-MDSCs and M-MDSCs, in ascites and spleens compared to the SAA1-KO group (Fig. 7D-G). Interestingly, although total CD4⁺ T cells were increased in the ascites of the SAA1-WT group, there were no significant differences in the overall CD4⁺ or CD8⁺ T cell populations in either spleens or ascites between the two groups (Fig. S5D-G). We therefore further examined the abundance of Treg cells and the effector function of CD8⁺ T cells. Treg cells were significantly higher in both spleens and ascites of the SAA1-WT group (Fig. 7H; Fig. S5H-I). Conversely, CD8⁺ T cells in the SAA1-KO group secreted significantly more IFN-γ and GZMB than those in the SAA1-WT group, in both spleens and ascites (Fig. 7I-J; Fig. S5J-M).

To elucidate the clinical significance of the SAA1/IL-1β/MDSC axis in EOC, we conducted a comprehensive evaluation of SAA1 expression and its correlation with IL-1β and CD33 levels in EOC tissues. Multiplex immunofluorescence (mIF) analysis revealed that elevated SAA1 expression in patients was closely associated with increased MDSCs infiltration and upregulated IL-1β expression (Fig. 7K). These findings were further validated in three independent datasets, which confirmed significant positive correlations between SAA1 expression and the levels of both CD33 and IL1B in ovarian cancer tissues (Fig. 7L–M). Moreover, patients with high expression of SAA1 and either CD33 or IL1B exhibited significantly shorter overall survival (OS) compared to controls (Fig. 7N–O). Notably, patients with concomitantly high levels of SAA1 and IL1B, along with a high proportion of CD33⁺ MDSCs, showed the poorest OS outcomes (Fig. 7P). Based on these prognostic factors, we developed a nomogram to quantitatively predict OS in patients with ovarian cancer (Fig. 7Q). These results underscore the strong clinical relevance of SAA1, IL1B and MDSCs infiltration as potent prognostic biomarkers in ovarian cancer.

Taken together, our research suggests that ovarian cancer cells secrete SAA1, which interacts with TLR2/4 on the surface of MDSCs, not only promoting MDSCs recruitment and GMP differentiation into MDSCs but also enhancing IL-1β secretion by MDSCs. In turn, IL-1β binds to IL-1R on ovarian cancer cells, activating the NF-κB pathway and further stimulating SAA1 production. This positive feedback loop drives immune evasion and accelerates the progression of ovarian cancer (Fig. 7R).

## Discussion

Ovarian cancer is one of the most common gynecological malignancies and has the highest mortality of all gynecological cancers [27]. Ovarian cancer development is a multifaceted process, wherein tumor cells exhibit heightened abilities in proliferation, migration, and invasion. Additionally, these cells possess the capability to regulate immune cells, shaping a tumor-promoting immunosuppressive microenvironment that facilitates immune escape and promotes ovarian cancer progression. Consequently, unveiling the malignant behavior of ovarian cancer cells and identifying key regulatory molecules involved in their interaction with immune cells is crucial for the diagnosis and treatment of ovarian cancer [28].

In recent decades, MDSCs have been increasingly recognized as a pivotal cell population within the innate immune system, exhibiting potent immunosuppressive activity in cancer and other pathological conditions. Studies have reported that MDSCs can promote Treg formation, mature into tumor-associated macrophages (TAMs), and drive the transition of fibroblasts into cancer-associated fibroblasts (CAFs) [29–32]. Consequently, MDSCs facilitate tumor immune evasion and diminish the efficacy of immunotherapy [33]. Moreover, circulating MDSC levels are positively correlated with advanced tumor stage and poor prognosis in patients [34]. In this study, we observed a significant increase in MDSCs, both M-MDSCs and PMN-MDSCs, in the peripheral blood and tumor tissues of patients with ovarian cancer. In parallel, the levels of their secreted immunosuppressive factors, including Arg-1, IDO and iNOS, were notably higher in tumor tissues. These findings suggest that MDSCs play a crucial role in ovarian cancer progression.

Building on this foundation, we delved deeper into the key molecular interactions between ovarian cancer cells and MDSCs. It was observed that SAA1 was markedly upregulated in ovarian cancer cells when co-cultured with MDSCs. Subsequent experiments validated that SAA1, at both the RNA and protein levels, exhibited substantial upregulation in ovarian cancer cells and tissues. Furthermore, its expression was significantly correlated with the FIGO stage of patients. Additional functional experiments demonstrated that SAA1 significantly enhanced the proliferation, migration, and invasion of ovarian cancer cells. Previous studies have indicated that EMT is a crucial process mediating tumor cell migration and invasion, regulating tumor cell stemness, and facilitating immune escape to adapt to the evolving tumor microenvironment [35, 36]. In our study, SAA1 was found to significantly affect the EMT pathway in ovarian cancer cells, which is consistent with a previous study revealing that arecoline induced EMT and promoted oral cancer metastasis through SAA1 expression [37]. Collectively, these experiments affirm that SAA1, despite being a secreted protein, plays a crucial role in sustaining the malignant phenotype of ovarian cancer cells.

SAA1 is acknowledged for its role as a cytokine-like protein facilitating cell-to-cell communication and feedback in inflammatory, immunological, tumor, and protective pathways [26]. Bioinformatics analyses indicate a robust correlation between SAA1 and the immunosuppressive microenvironment in ovarian cancer patients, with a primary focus on MDSCs. However, studies examining whether tumor cells contribute to tumor immune escape by regulating MDSCs through SAA1 are currently lacking. MDSCs undergo two stages to exert their functions. The first stage involves the expansion and aggregation of MDSCs, while the second stage involves the activation of MDSCs into cells with immunosuppressive functions, driven by continuous tumor-derived signals [38]. Thus, ovarian cancer cells were co-cultured with MDSCs to investigate the impact of SAA1 on MDSCs recruitment and activation. Results revealed that the knockdown of SAA1 in ovarian cancer cells significantly diminished the ability of cell supernatants to recruit MDSCs. Simultaneously, the capacity to promote the differentiation of GMPs into MDSCs was notably inhibited. Subsequently, we delved into the specific mechanism by which SAA1 regulates MDSCs. Earlier studies have clarified that the receptors of SAA1 primarily include TLR2/4, FPR2, etc. Notably, TLR2/4, key receptors on the surface of MDSCs, play a pivotal role in the recruitment and activation of MDSCs [16, 39, 40]. Consequently, we chose TLR2/4 for further investigation. Following the determination of TLR2/4 expression on the surface of MDSCs using flow cytometry, a series of rescue experiments were conducted using recombinant SAA1 protein and the TLR2/4 antagonist SsnB. The results confirmed that SAA1 released from ovarian cancer cells indeed promotes the recruitment and activation of MDSCs through its interaction with TLR2/4 on the surface of MDSCs. These experiments confirm that SAA1 released by ovarian cancer cells acts on TLR2/4 on the surface of MDSCs, thereby promoting MDSCs recruitment and the differentiation of GMPs into MDSCs.

Previous studies have demonstrated that SAA1, an acute-phase inflammatory protein, enhances the secretion of cytokines including IL-1β, IL-6, and TNF-α by immune cells. As immunosuppressive cells, MDSCs also secrete various cytokines, particularly immunosuppressive factors such as Arg-1, iNOS, and IDO [26]. Our study reveals that SAA1 regulates IL-1β secretion by MDSCs through TLR2/4, while the secreted IL-1β in turn stimulates SAA1 production in ovarian cancer cells, thereby establishing a positive feedback loop between these components. IL-1β, a pro-inflammatory cytokine primarily secreted by monocytes, macrophages, and dendritic cells, is known to activate NF-κB and MAPK signaling pathways within the tumor microenvironment [41]. These pathways promote critical tumorigenic processes including cellular proliferation, invasion, angiogenesis, and immune evasion [42]. Our findings demonstrate that IL-1β activates the NF-κB pathway via IL-1R, facilitating P65 transcription factor binding to the SAA1 promoter region and consequent upregulation of SAA1 expression. This mechanistic insight reveals how the SAA1-IL-1β feedback loop between ovarian cancer cells and MDSCs contributes to immune evasion. In vivo validation using a C57BL/6 mouse peritoneal tumor model confirmed that SAA1 promotes the development of a tumor-promoting immunosuppressive microenvironment that facilitates the formation of ovarian cancer-associated ascites.

While this study, for the first time, uncovers that ovarian cancer cells and MDSCs form a positive feedback loop through the SAA1-IL-1β axis to promote immune evasion, thereby providing a new target for ovarian cancer diagnosis and treatment, there are still some limitations. Firstly, despite SAA1 being a secreted protein, it remains unclear whether it can regulate the secretion of other cytokines by ovarian cancer cells, thereby influencing MDSCs. Secondly, the intracellular signaling alterations in MDSCs following SAA1-TLR2/4 interaction need detailed characterization. Lastly, the therapeutic efficacy of targeting SAA1 and IL-1β in ovarian cancer immunotherapy warrants further comprehensive investigation.

## Conclusions

In conclusion, this study elucidates the multifaceted tumor-promoting role of SAA1 in the pathogenesis of ovarian cancer. SAA1 directly enhances key malignant behaviors of ovarian cancer cells, including proliferation, migration, and invasion. In addition, cancer cell–derived SAA1 promotes the recruitment of MDSCs and their differentiation from GMPs via TLR2/4 signaling, while also inducing IL-1β secretion by MDSCs. This secreted IL-1β further amplifies SAA1 expression in ovarian cancer cells through IL-1R/NF-κB signaling, thereby forming a positive feedback loop that reinforces a tumor-promoting immunosuppressive microenvironment conducive to immune evasion. Consistent with these mechanistic insights, clinical analyses revealed that elevated levels of SAA1, IL1B, and CD33⁺ MDSCs were significantly correlated with poorer overall survival in EOC patients, underscoring the SAA1/IL-1β/MDSC axis as a key driver of tumor progression and adverse prognosis. These findings not only establish SAA1 as a promising therapeutic target but also provide valuable direction for the development of novel immunotherapeutic strategies against ovarian cancer.

## Supplementary Information

Below is the link to the electronic supplementary material.

Supplementary Material 1: Figure S1 to S5 and Tables S1 to S7

## Data Availability

All data supporting the findings of this study are available within the manuscript and its supplementary information files.

## Abbreviations

EOC: Epithelial ovarian cancer
MDSCs: Myeloid-derived suppressor cells
PMN-MDSCs: Polymorphonuclear MDSCs
M-MDSCs: Monocyte MDSCs
SAA1: Serum amyloid A1
GMPs: Granulocyte-monocyte progenitors
TME: Tumor microenvironment
IMCs: Immature myeloid cells
ROS: Reactive oxygen species
NO: Nitric oxide
Arg-1: Arginase-1
Tregs: Regulatory T cells
PBMCs: Peripheral blood mononuclear cells
qRT-PCR: quantitative Real-time polymerase chain reaction
JASPAR: Joint Annotation of Sequence motifs and Protein Regulatory elements
GTRD: Gene Transcription Regulation Database
Human TFDB: Human Transcription Factor Database
ChIP: Chromatin Immunoprecipitation
IHC: Immunohistochemistry
shRNA: Short hairpin RNA
CCK8: Cell Counting Kit-8
GEO: Gene Expression Omnibus
GEPIA: Gene Expression Profiling Interactive Analysis
TCGA: The Cancer Genome Atlas
TIMER: Tumor Immune Estimation Resource
TAMs: Tumor-associated macrophages
CAFs: Cancer-associated fibroblasts
SsnB: Sparstolonin B
IL-1β: Interleukin-1beta
