# Supplementary material for: Tumor cells promote immunosuppression in ovarian cancer via a positive feedback loop with MDSCs through the SAA1–IL-1β axis
Source: J Exp Clin Cancer Res. 2025 Sep 30;44:277. doi: 10.1186/s13046-025-03536-y (PMC12486657; doi:10.1186/s13046-025-03536-y)
Supplement: Supplementary file 1 — Supplementary Material 1: Figure S1 to S5 and Tables S1 to S7 [file 13046_2025_3536_MOESM1_ESM.docx]

**Supplementary Materials for**

**Tumor Cells Promote Immunosuppression in Ovarian Cancer via a Positive Feedback Loop with MDSCs through the SAA1–IL-1β Axis**

Haoran Hu et al.

Corresponding Author: Han Lingfei, lingfeihan@tongji.edu.cn; Wang Wei, 2405132@tongji.edu.cn; Li Ang, [liang@tongji.edu.cn](mailto:liang@tongji.edu.cn)

**The file includes:**

Figs.S1 to S5

Tables S1 to S7

**
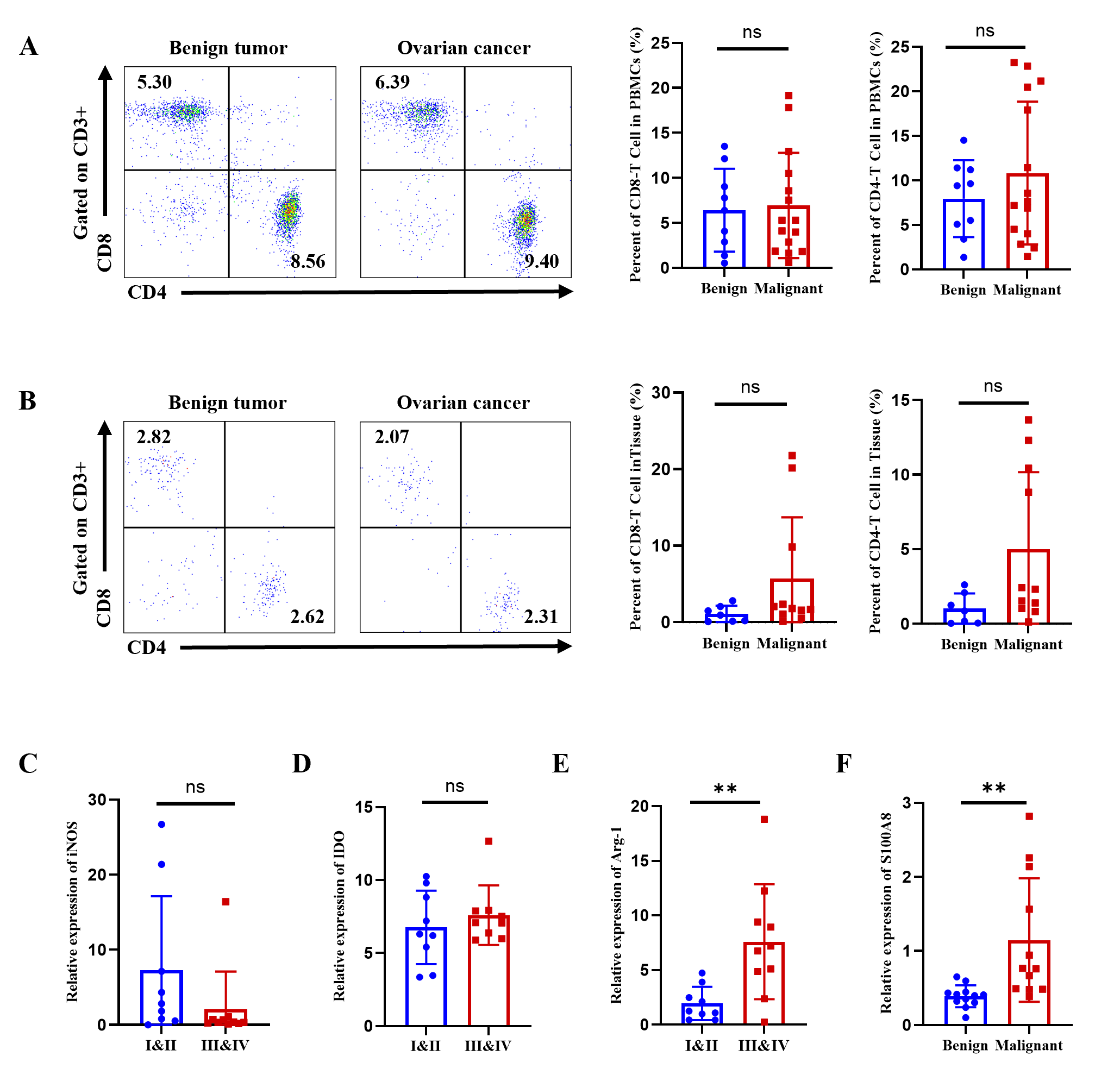
**

**Supplementary Figure. 1** Distribution of immune cells and immunosuppressive factors in ovarian cancer. (**A**) Flow cytometry analysis of CD4⁺ and CD8⁺ T cells in PBMCs from patients with benign or malignant ovarian tumors. Each dot represents an individual patient; data are presented as mean ± SEM; Mann–Whitney U test. (**B**) Flow cytometry analysis of CD4⁺ and CD8⁺ T cells in tumor-infiltrating lymphocytes from patients with benign or malignant ovarian tumors. Each dot represents an individual patient; data are presented as mean ± SEM; Mann–Whitney U test. (**C-E**) qRT-PCR analysis of immunosuppressive factors iNOS, IDO, and Arg-1 in tumor tissues from patients at different FIGO stages. Each dot represents an individual patient; data are presented as mean ± SEM; Mann–Whitney U test. (**F**) qRT-PCR analysis of S100A8 expression in tumor tissues from patients with benign or malignant ovarian tumors. Each dot represents an individual patient; data are presented as mean ± SEM; Mann–Whitney U test. *Statistical significance: ***P* < 0.01; ns, not significant.

**
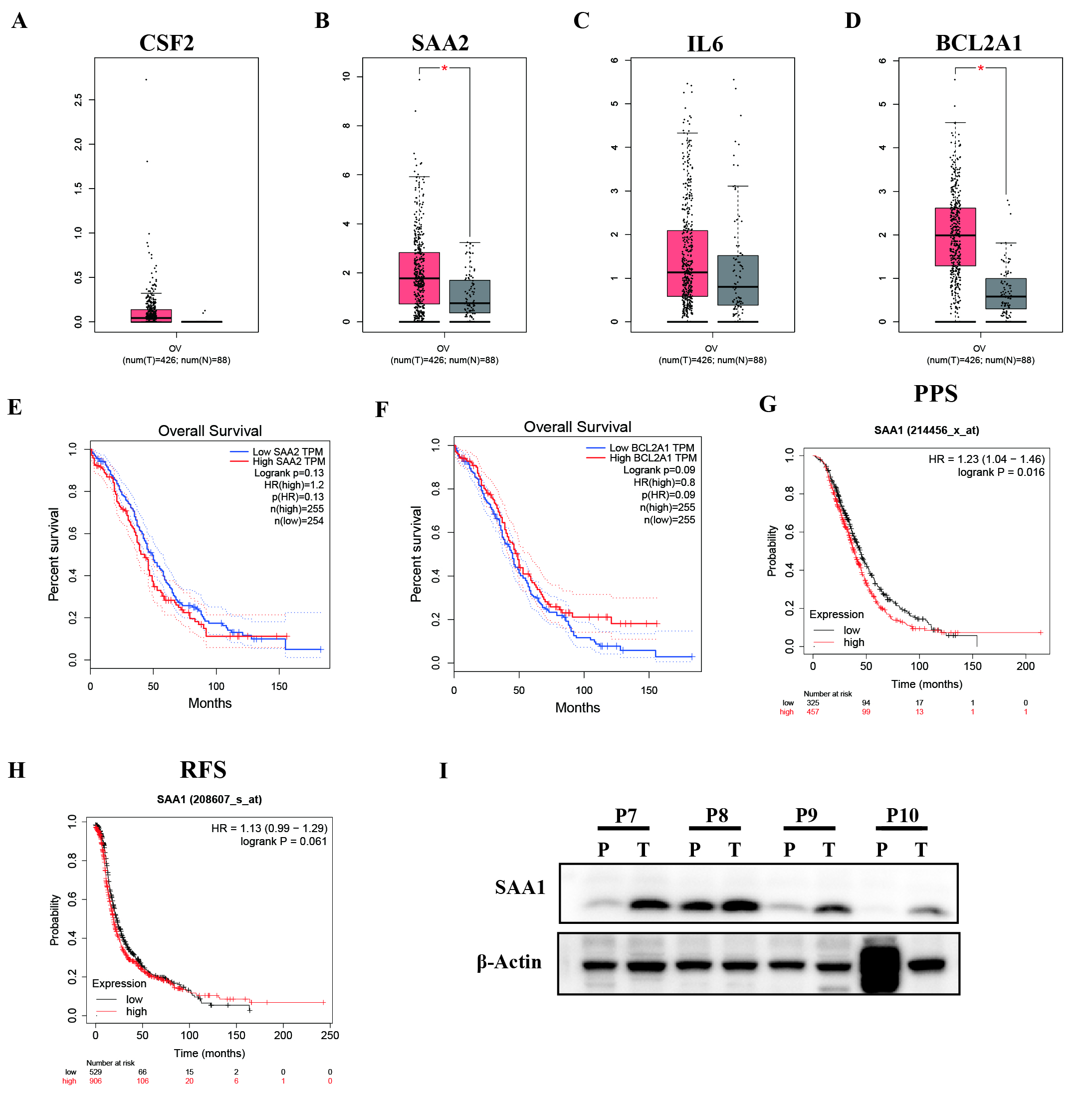
**

**Supplementary Figure. 2** SAA1 is elevated and associated with poor prognosis in EOC patients. **(A)** CSF2 expression in ovarian cancer tissues based on the GEPIA database. Each dot represents one sample; data are presented as mean ± SEM; unpaired two-tailed Student’s t-test. **(B)** SAA2 expression in ovarian cancer tissues based on the GEPIA database. Each dot represents one sample; data are presented as mean ± SEM; unpaired two-tailed Student’s t-test. **(C)** IL6 expression in ovarian cancer tissues based on the GEPIA database. Each dot represents one sample; data are presented as mean ± SEM; unpaired two-tailed Student’s t-test. **(D)** BCL2A1 expression in ovarian cancer tissues based on the GEPIA database. Each dot represents one sample; data are presented as mean ± SEM; unpaired two-tailed Student’s t-test. **(E)** Kaplan–Meier analysis of overall survival in ovarian cancer patients stratified by SAA2 expression using GEPIA. Log-rank test. **(F)** Kaplan–Meier analysis of overall survival in ovarian cancer patients stratified by BCL2A1 expression using GEPIA. Log-rank test. **(G)** Kaplan–Meier analysis of PPS in ovarian cancer patients stratified by SAA1 expression using the Kaplan–Meier Plotter database. Log-rank test. **(H)** Kaplan–Meier analysis of RFS in ovarian cancer patients stratified by SAA1 expression using the Kaplan–Meier Plotter database. Log-rank test. (**I**) Detection of SAA1 protein expression in paired tumor (T) and adjacent paracancerous (P) tissues from EOC patients by Western blotting. *Statistical significance: **P* < 0.05; ns, not significant.


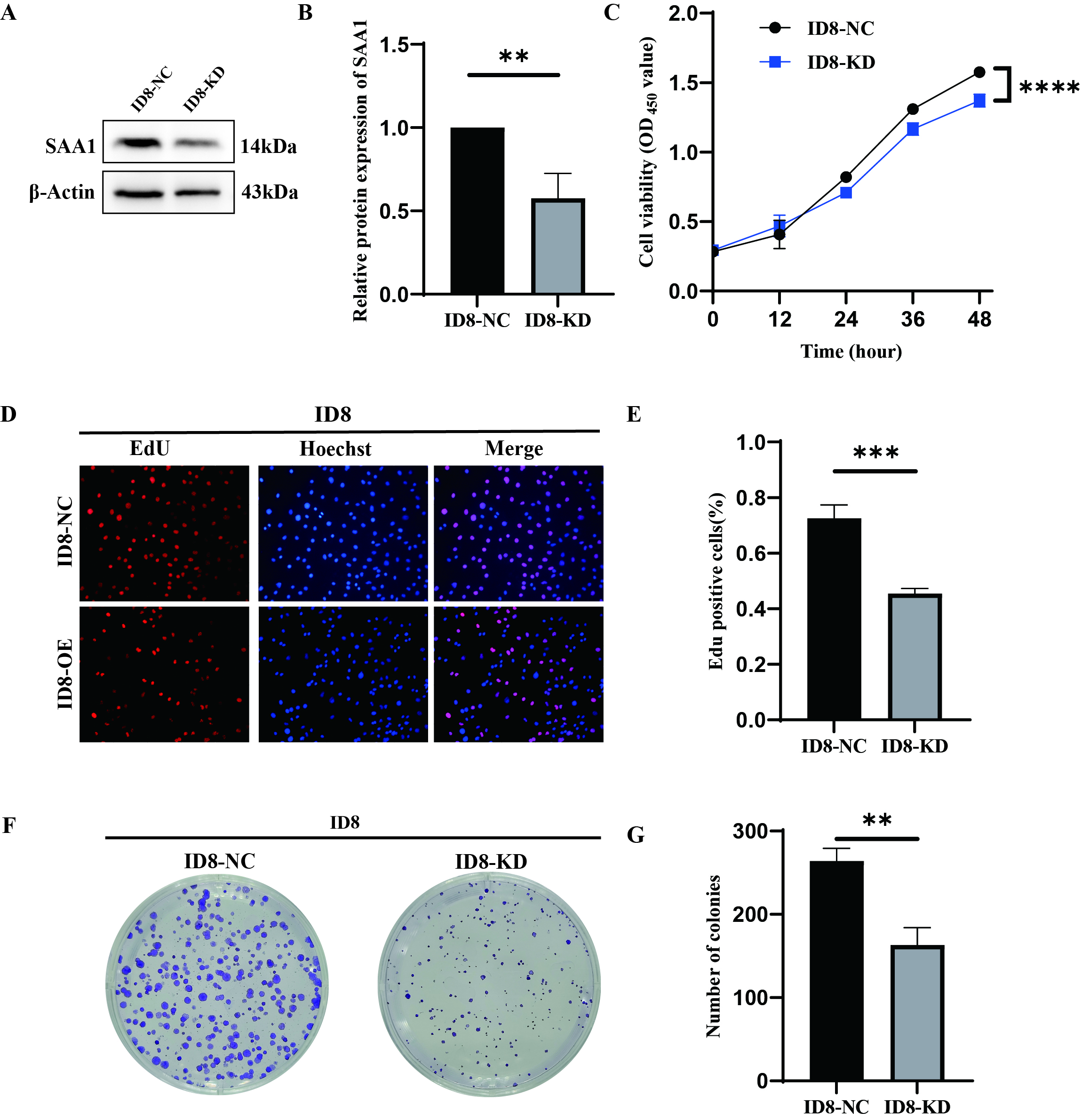


**Supplementary Figure. 3** SAA1 promotes the proliferation of mouse-derived ovarian cancer cells in vitro. **(A–B)** Detection of SAA1 knockdown efficiency at the protein level in ID8 cells by Western blotting (A) and densitometric analysis (B). Data are presented as mean ± SEM from three independent experiments; unpaired two-tailed Student’s t-test. **(C)** CCK8 assay to evaluate the effect of SAA1 knockdown on ID8 cell proliferation. Data are presented as mean ± SEM from three independent experiments; unpaired two-tailed Student’s t-test. **(D–E)** EdU assay to evaluate the proliferative capacity of ID8 cells following SAA1 knockdown. Data are presented as mean ± SEM from three independent experiments; unpaired two-tailed Student’s t-test. **(F–G)** Colony formation assay to assess the clonogenic capacity of ID8 cells following SAA1 knockdown. Data are presented as mean ± SEM from three independent experiments; unpaired two-tailed Student’s t-test. *Statistical significance: ***P* < 0.01; ****P* < 0.001; *****P* < 0.0001.


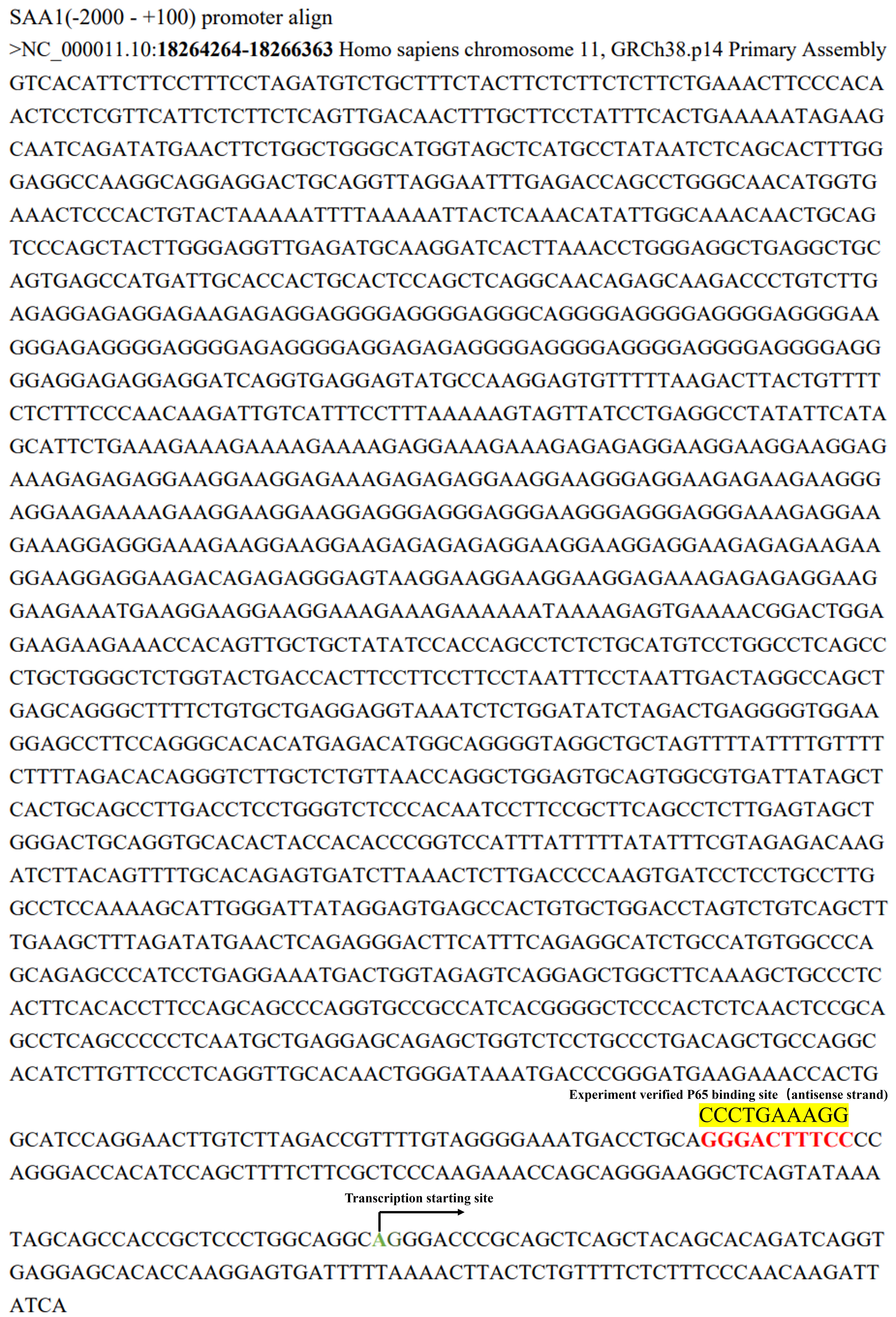


**Supplementary Figure. 4** The sequence of SAA1 promoter. The P65 binding sites are marked in yellow and the transcription start site is marked in green.


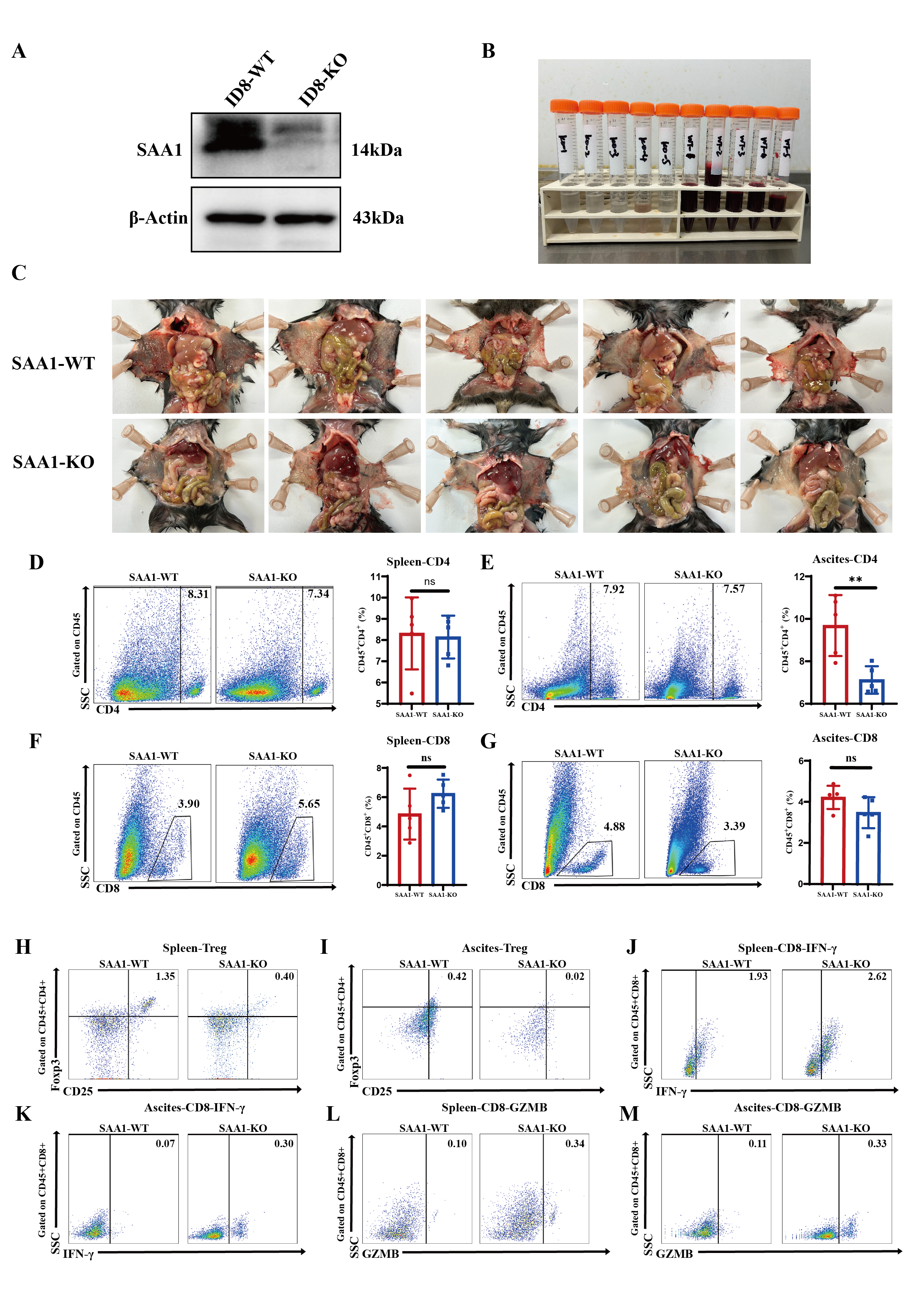


**Supplementary Figure. 5** SAA1 promotes ovarian cancer immunosuppression leading to ascites formation in vivo. (**A**) Detection of SAA1 knockout efficiency in ID8 cells by Western blotting. (**B**) Representative images showing differences in ascites formation between SAA1-WT and SAA1-KO mice. (**C**) Representative gross images showing differences in tumor nodule formation on the abdominal wall between SAA1-WT and SAA1-KO mice. (**D-E**) Flow cytometry analysis of the total CD4⁺ T cell population in the spleen (D) and ascites (E) of SAA1-WT and SAA1-KO mice, with corresponding quantification. Each dot represents one mouse; data are presented as mean ± SEM; unpaired two-tailed Student’s t-test. **(F–G)** Flow cytometry analysis of the total CD8⁺ T cell population in the spleen (F) and ascites (G) of SAA1-WT and SAA1-KO mice, with corresponding quantification. Each dot represents one mouse; data are presented as mean ± SEM; unpaired two-tailed Student’s t-test. **(H–I)** Flow cytometry analysis of Treg cells in the spleen (H) and ascites (I) of SAA1-WT and SAA1-KO mice. **(J–K)** Flow cytometry analysis of the ability of CD8⁺ T cells to secrete IFN-γ in the spleen (J) and ascites (K) of SAA1-WT and SAA1-KO mice. **(L–M)** Flow cytometry analysis of the ability of CD8⁺ T cells to secrete GZMB in the spleen (L) and ascites (M) of SAA1-WT and SAA1-KO mice. *Statistical significance: ***P* < 0.01; ns, not significant.

| **Supplementary Table S1.** Baseline Clinicopathological Characteristics of Study Participants. | | | | |
| --- | --- | --- | --- | --- |
| **Clinical Characteristics** | **Control Group**  **(n=17)** |  | **Ovarian Cancer Group(n=49)** | ***P*-value** |
| **Age (years)** | 63 (55–68) |  | 54 (48–64) | 0.023* |
| **Histological Type** |  |  |  |  |
| Pelvic organ prolapse^1^ | 7 (41.2%) | High-grade serous carcinoma | 27 (55.1%) |  |
| Uterine leiomyoma | 5 (29.4%) | Clear cell carcinoma | 11 (22.4%) |  |
| Cervical HSIL | 4 (23.5%) | Endometrioid carcinoma | 4 (8.2%) |  |
| Adenomyosis | 1 (5.9%) | Others^2^ | 7 (14.3%) |  |
| **FIGO stage** |  |  |  |  |
| I | / |  | 9 (18.4%) |  |
| II | / |  | 11 (22.4%) |  |
| III | / |  | 24 (49.0%) |  |
| IV | / |  | 5 (10.2%) |  |
| **Tumor Grade** |  |  |  |  |
| G1 | / |  | 5 (10.2%) |  |
| G2 | / |  | 18 (36.7%) |  |
| G3 | / |  | 26 (53.1%) |  |
| **CA125 (U/mL)** | 9.6（8.1–11.2） |  | 143.9（74.1–504.2） | 2.38×10⁻⁷* |
| **Menopausal Status** |  |  |  |  |
| Premenopausal | 2 (11.8%) |  | 15 (30.6%) |  |
| Postmenopausal | 15 (88.2%) |  | 34 (69.4%) |  |

FIGO: International Federation of Gynecology and Obstetrics; HSIL: High-grade squamous intraepithelial lesion; **P*<0.05

**Non-parametric Mann–Whitney U test**.

^1^ Pelvic organ prolapse includes uterine prolapse, anterior and/or posterior vaginal wall prolapse, as clinically diagnosed.

^2^ Others include low-grade serous carcinoma, mucinous carcinoma, borderline tumors and other rare subtypes.

**Supplementary Table S2. Antibodies used for flow cytometry.**

| **Antibodies** | **Source** | **Cat. No.** |
| --- | --- | --- |
| FITC anti-human CD11b antibody | Biolegend | 301330 |
| APC anti-human CD33 antibody | Biolegend | 366606 |
| PerCP/Cyanine5.5 anti-human HLA-DR antibody | Biolegend | 307630 |
| PerCP/Cyanine5.5 anti-human CD14 antibody | Biolegend | 392305 |
| PE anti-human CD15 antibody | Biolegend | 301,906 |
| Brilliant Violet 421 anti-human CD15 antibody | Biolegend | 323039 |
| PE anti-human CD3 antibody | Biolegend | 317308 |
| APC anti-human CD4 antibody | Biolegend | 300537 |
| FITC anti-human CD8 antibody | BD Bioscience | 555634 |
| FITC anti-human CD25 antibody | BD Bioscience | 555431 |
| PE anti-human FOXP3 antibody | BD Bioscience | 555431 |
| PE anti-human TLR2 antibody | Biolegend | 392305 |
| PE anti-human TLR4 antibody | Biolegend | 312805 |
| Brilliant Violet 421TM anti-mouse CD45 antibody | Biolegend | 103134 |
| APC anti-mouse/human CD11b antibody | Biolegend | 101212 |
| PerCP/Cyanine5.5 anti-mouse Ly6G antibody | Biolegend | 127615 |
| Alexa Fluor® 488 anti-mouse Ly6C antibody | Biolegend | 128021 |
| APC anti-mouse CD4 antibody | Biolegend | 100412 |
| FITC anti-mouse CD25 antibody | Biolegend | 102005 |
| PE anti-mouse FOXP3 antibody | Biolegend | 126403 |
| PerCP/Cyanine5.5 anti-mouse CD8a antibody | Biolegend | 100734 |
| Brilliant Violet 510TM anti-mouse IFN-γ antibody | Biolegend | 505841 |
| PE anti-mouse GZMB antibody | Biolegend | 396405 |

**Supplementary Table S3. Primers Sequences for qRT-PCR**

| **Gene** | **Species** | **Forward Primer** | **Reverse Primer** |
| --- | --- | --- | --- |
| iNOS | Human | GTTCTCAAGGCACAGGTCTC | GCAGGTCACTTATGTCACTTATC |
| IDO | Human | GCCCTTCAAGTGTTTCACCAA | CCAGCCAGACAAATATATGCGA |
| Arg-1 | Human | TGGACAGACTAGGAATTGGCA | CCAGTCCGTCAACATCAAAACT |
| SAA1 | Human | CATGCTCGGGGGAACTAT | TACCCATTGTGTACCCTCTCC |
| S100A8 | Human | CTTCCAGGAGTTCCTCATTCTG | AGCTACTCTTTGTGGCTTTCT |
| GAPDH | Human | CTGACTTCAACAGCGACACC | TGCTGTAGCCAAATTCGTTGT |

**Supplementary Table S4. Antibodies used for Western Blot.**

| **Antibodies** | **Source** | **Cat. No.** |
| --- | --- | --- |
| Anti-human IκBα antibody | Cell Signaling Technology | #9242 |
| Anti-human P-IκBα antibody | Cell Signaling Technology | #2859 |
| Anti-human P65 antibody | Cell Signaling Technology | #8242 |
| Anti-human P-P65 antibody | Cell Signaling Technology | #3033 |
| Anti-human SAA1 antibody | Abcam | ab207445 |
| Anti-mouse SAA1 antibody | Abcam | ab199030 |
| Anti-human/mouse E-cadherin antibody | ABclonal | A3044 |
| Anti-human/mouse N-cadherin antibody | ABclonal | A3045 |
| Anti-human/mouse Vimentin antibody | ABclonal | A19607 |
| Anti-human/mouse Snail antibody | ABclonal | A5243 |
| Anti-human/mouse β-Actin antibody | Servicebio | GB11001 |
| HRP conjugated Goat Anti-Rabbit IgG (H+L) | Servicebio | GB23303 |

**Supplementary Table S5. shRNA and sgRNA sequence used in this study.**

| **Gene** | **Species** | **Sequence** | **Source** |
| --- | --- | --- | --- |
| shSAA1-1 | Human | CCAGAGAGAATATCCAGAGAT | Tsingke Biotech |
| shSAA1-2 | Human | CCAGAGAGAATATCCAGAGAT | Tsingke Biotech |
| shSAA1-3 | Human | CCAGAGAGAATATCCAGAGAT | Tsingke Biotech |
| shSAA1 | Mouse | TCATTTGTTCACGAGGCTTTC | Geneseed |
| sgSAA1 | Mouse | GCTGCTCAAAGGGGTCCCGG | Zhang Lab |

**Supplementary Table S6. ChIP primers used in this study.**

| **Gene** | **Species** | **5’-Forward Primer-3’** | **5’-Reverse Primer-3’** |
| --- | --- | --- | --- |
| SAA1 | Human | CTCAGGTTGCACAACTGGGATA | GCTATTTATACTGAGCCTTCCCTGC |

**Supplementary Table S7.** **Levels of immune cell subsets in peripheral blood and tumor tissue of patients.**

| **Immunocyte** | **Peripheral Blood** | |  |  | **Tumor Tissue** | |  |
| --- | --- | --- | --- | --- | --- | --- | --- |
|  | **Benign** | **Malignant** | **P** |  | **Benign** | **Malignant** | **P** |
| M-MDSC | 3.691±2.911 | 7.608±3.943 | **0.008**** |  | 0.289±0.360 | 5.592±6.405 | **0.012*** |
| PMN-MDSC | 1.446±1.350 | 3.109±2.262 | **0.036*** |  | 0.511±0.592 | 4.540±4.497 | **0.007**** |
| CD4^+^T Cells | 7.944±4.310 | 10.83±8.021 | 0.332 |  | 1.105±1.031 | 4.992±5.180 | 0.065 |
| CD8^+^T Cells | 6.412±4.608 | 6.935±5.845 | 0.822 |  | 1.102±1.064 | 5.722±7.981 | 0.151 |
| Treg cells | 1.408±0.820 | 3.377±1.440 | **0.029*** |  |  |  |  |

Non-parametric Mann–Whitney U test.
